# The use of antibiotic, probiotic, and fecal microbiota transplantation in modulating immunotherapy efficacy and survival: a systematic review and meta-analysis of clinical outcomes

**DOI:** 10.1093/oncolo/oyag264

**Published:** 2026-07-10

**Authors:** Zijun Zhai, Shuling Ma, Shijie Shang, Xinyi Liang, Shan Yin, Haofeng Lin, Rui Ding, Aimin Jiang, Ran Zhang, Meng Wu, Jinming Yu, Qian Song, Dawei Chen

**Affiliations:** Cheeloo College of Medicine, Shandong University Cancer Center, Jinan, 250000, China; Department of Shandong Provincial Key Laboratory of Precision Oncology, Shandong Cancer Hospital and Institute, Shandong First Medical University and Shandong Academy of Medical Sciences, Jinan, 250117, China; Department of Shandong Provincial Key Laboratory of Precision Oncology, Shandong Cancer Hospital and Institute, Shandong First Medical University and Shandong Academy of Medical Sciences, Jinan, 250117, China; Department of Shandong Provincial Key Laboratory of Precision Oncology, Shandong Cancer Hospital and Institute, Shandong First Medical University and Shandong Academy of Medical Sciences, Jinan, 250117, China; Cancer Center, Union Hospital, Tongji Medical College, Huazhong University of Science and Technology, Wuhan, 430023, China; Department of Shandong Provincial Key Laboratory of Precision Oncology, Shandong Cancer Hospital and Institute, Shandong First Medical University and Shandong Academy of Medical Sciences, Jinan, 250117, China; Cheeloo College of Medicine, Shandong University Cancer Center, Jinan, 250000, China; Department of Shandong Provincial Key Laboratory of Precision Oncology, Shandong Cancer Hospital and Institute, Shandong First Medical University and Shandong Academy of Medical Sciences, Jinan, 250117, China; Guangdong Provincial Key Laboratory of Pharmaceutical Bioactive Substances, Guangdong Pharmaceutical University, Guangzhou, 510006, People’s Republic of China; Department of Shandong Provincial Key Laboratory of Precision Oncology, Shandong Cancer Hospital and Institute, Shandong First Medical University and Shandong Academy of Medical Sciences, Jinan, 250117, China; Department of Shandong Provincial Key Laboratory of Precision Oncology, Shandong Cancer Hospital and Institute, Shandong First Medical University and Shandong Academy of Medical Sciences, Jinan, 250117, China; Department of Shandong Provincial Key Laboratory of Precision Oncology, Shandong Cancer Hospital and Institute, Shandong First Medical University and Shandong Academy of Medical Sciences, Jinan, 250117, China; Department of Shandong Provincial Key Laboratory of Precision Oncology, Shandong Cancer Hospital and Institute, Shandong First Medical University and Shandong Academy of Medical Sciences, Jinan, 250117, China; Department of Shandong Provincial Key Laboratory of Precision Oncology, Shandong Cancer Hospital and Institute, Shandong First Medical University and Shandong Academy of Medical Sciences, Jinan, 250117, China; Department of Shandong Provincial Key Laboratory of Precision Oncology, Shandong Cancer Hospital and Institute, Shandong First Medical University and Shandong Academy of Medical Sciences, Jinan, 250117, China; Cheeloo College of Medicine, Shandong University Cancer Center, Jinan, 250000, China; Department of Shandong Provincial Key Laboratory of Precision Oncology, Shandong Cancer Hospital and Institute, Shandong First Medical University and Shandong Academy of Medical Sciences, Jinan, 250117, China

**Keywords:** antibiotic, probiotic, fecal microbiota transplantation, microbiota, immune checkpoint inhibitors

## Abstract

**Background:**

Immune checkpoint inhibitors (ICIs) have been one of the important therapeutic approaches for patients with advanced malignancies; nevertheless, their clinical efficacy remains limited in many patients. Recently, the contribution of intestinal microbiota to improved antitumor immune responses has gradually been recognized.

**Methods:**

A comprehensive literature search was conducted in PubMed, Embase, and the Cochrane Library to identify relevant studies published up to June 15, 2026. We evaluated the influence of microbiota interventions with respect to efficacy and survival in cancer patients receiving ICIs from 3 perspectives: antibiotics, probiotics, and fecal microbiota transplantation (FMT). The main endpoint was objective response rate (ORR), and secondary endpoints were overall survival (OS) and progression-free survival (PFS).

**Results:**

The final analysis comprised 106 studies, which were categorized into 3 groups: antibiotics (76 studies), probiotics (15 studies), and FMT (15 studies). Antibiotic use was correlated with compromised immunotherapy efficacy and unfavorable survival outcomes. In particular, antibiotic exposure was linked to a reduced ORR (odds ratio [OR] = 0.60, 95% CI, 0.46-0.77, *P* < .001), shorter OS (hazard ratio [HR] = 1.56, 95% CI, 1.44-1.69, *P* < .001), and shorter PFS (HR = 1.50, 95% CI, 1.32-1.70, *P* < .001). In contrast, probiotics showed a supportive and positive effect on immunotherapy outcomes, with improved ORR (OR = 1.95, 95% CI, 1.46-2.62, *P* < .001) and better OS (HR = 0.56, 95% CI, 0.41-0.78, *P* < .001) and PFS (HR = 0.53, 95% CI= 0.38-0.74, *P* < .001). FMT combined with immunotherapy achieved a favorable ORR of 0.30 (95% CI, 0.16-0.45, *P* < .001).

**Conclusions:**

This meta-analysis synthesized evidence from studies on antibiotics, probiotics, and FMT use, suggesting gut microbiota offering potential approaches to enhance immunotherapy treatment effectiveness and clinical efficacy in individuals with advanced-stage solid cancers.

Implications for Practice This meta-analysis indicates that gut microbiota modulation may represent a clinically relevant factor influencing the efficacy of immune checkpoint inhibitors across advanced solid tumors. Antibiotic exposure was consistently associated with reduced treatment response and inferior survival, supporting the need for careful antibiotic stewardship during immunotherapy whenever clinically feasible. In contrast, probiotics and fecal microbiota transplantation showed encouraging signals of benefit, although the current evidence remains insufficient to justify routine implementation outside prospective trials or selected investigational settings. Collectively, these findings underscore the microbiome as a potentially actionable therapeutic target and provide a rationale for integrating microbiota-aware strategies into immuno-oncology research and future clinical decision-making.

## Introduction

Immune checkpoint inhibitors (ICIs) have markedly revolutionized therapeutic approaches of multiple malignancies. However, their clinical efficacy remains limited in many patients, emphasizing an urgent need to identify modifiable biological factors to enhance therapeutic efficacy and improve survival outcomes. Recently, research supports the notion that the gut microbiota contributes to determining ICI responsiveness and resistance by modulating antigen presentation, the activity of effector T cells together with immune-inflammatory mechanisms. These underscore the “microbiota-immunotherapy axis” as an important frontier in immuno-oncology research.^1^

Antibiotics can rapidly reduce microbial diversity and alter metabolic pathways and have therefore been widely used to determine the influence of “microbiota disruption” on ICI endpoints.^2^ Recent clinical studies have suggested that antibiotic exposure within specific time windows before or after ICIs was related to worse outcome. However, the heterogeneity in the definitions of antibiotic exposure—including antibiotic class, duration, timing, and prophylactic versus therapeutic use—may contribute to substantial between-study variability.^2^ Therefore, studies with more rigorous designs and finer stratification of antibiotic exposure are still needed to clarify the magnitude and sensitive time windows of antibiotic effects across different cancer types and treatment regimens, and to provide more actionable evidence for antimicrobial stewardship in ICIs.^2^

In contrast, probiotics, as a relatively “mild” microbiota intervention strategy, may arise their effects through mechanisms including improving the intestinal mucosal barrier, increasing the production of immunostimulatory metabolites, and modulating antigen presentation and T-cell effector functions.^1^ In recent years, some clinical trials have examined the associations between probiotic use and ICI’s efficacy and survival. For example, in melanoma, exploratory randomized, placebo-controlled, stratified trials of microbiome-based consortia products (eg, SER-401) suggested feasibility in terms of safety, immune modulation, and microbiome plasticity.^3^ However, substantial heterogeneity in formulations (single-strain products, multi-strain products, and microbial consortia), small sample size, and diverse endpoints collectively limit reproducibility and generalizability.^4^ As a more advanced microbiota-modulating approach, fecal microbiota transplantation (FMT) has been expected to reverse immune tolerance or restore sensitivity in patients with ICIs.^1^ FMT has entered early-stage clinical validation in ICI-resistant populations. For example, the phase 1 MIMic trial reported clinical outcomes and feasibility data for combination of FMT with anti-PD-1 therapy in individuals with advanced melanoma. Meanwhile, preliminary findings from the phase 2 randomized TACITO trial suggested that adding FMT to pembrolizumab plus axitinib may enhance therapeutic efficacy in metastatic renal cell cancer (RCC).^5^

Based on the above advances, the 3 categories of microbiota-related exposures or interventions can be viewed as forming a biological continuum: antibiotics for “microbiota disruption”, probiotics for “microbiota support or supplementation”, and FMT for “microbiota reconstruction”. However, existing studies are highly heterogeneous in terms of study design, exposure definitions, cancer types, and treatment lines, indicating that clinical conclusions still require systematic integration.^1^ These limitations highlight the necessity to comprehensively analyze relation between microbiota and immunotherapy.

This meta-analysis provides a comprehensive evaluation of the associations of antibiotics, probiotics, and FMT focusing on therapeutic efficacy and survival, using objective response rate (ORR), overall survival (OS), and progression-free survival (PFS) as endpoints. In addition, sources of heterogeneity—such as line of therapy, cancer types, treatment regimens, trial designs, and immunotherapeutic agents—were explored to provide a more actionable evidence framework for antimicrobial stewardship, selection of microbiota-based adjunctive therapies, and the design of future randomized trials.

## Methods

This study compiled clinical data from patients receiving ICIs in combination with antibiotics, probiotics, or FMT, aiming to provide a comprehensive evaluation of patient outcomes of these combination strategies. The research followed the Preferred Reporting Items for Systematic Reviews and Meta-Analyses (PRISMA) guidelines (PROSPERO, CRD420251238409). As this study was based solely on de-identified patient data, ethical approval and consent were not required.

### Data sources and literature search methods

**Figure 1.**
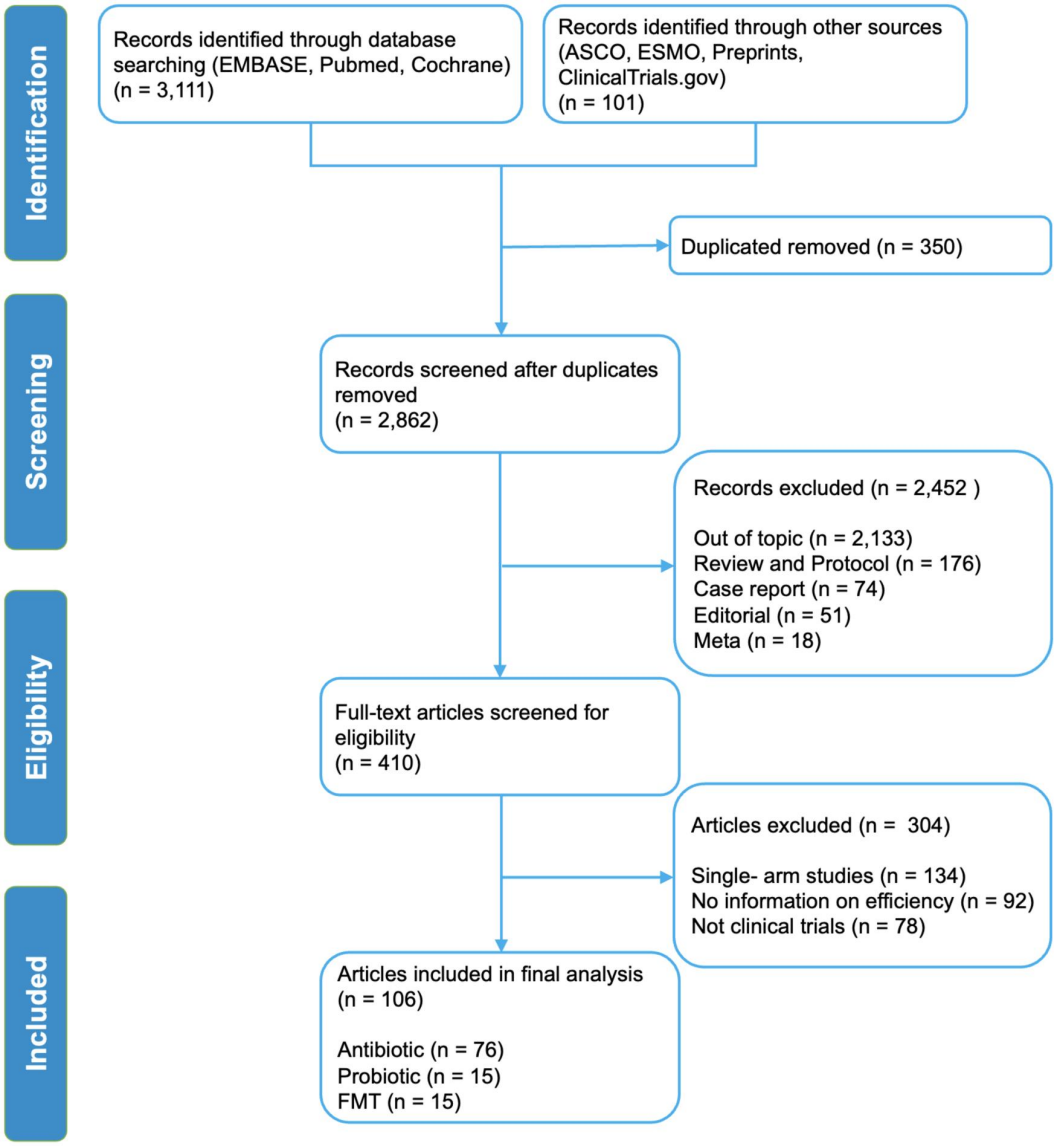
PRISMA flow diagram showing the identification, screening, eligibility assessment, and inclusion of studies. Ultimately, 106 studies were included: 76 antibiotic studies, 15 probiotic studies, and 15 FMT studies. FMT, fecal microbiota transplantation..

Published literature of EMBASE, PubMed, and Cochrane Library were systematically searched including articles available online up to June 15, 2026. The detailed search strategies are provided in Tables S1-S3. Language and publication type were not restricted, and the initial search yielded a total of 3111 records. In addition, we comprehensively searched ClinicalTrials.gov, preprint platforms, and abstracts from the American Society of Clinical Oncology (ASCO) and the European Society for Medical Oncology (ESMO), using “antibiotics,” “probiotics,” “FMT,” as well as cancer-related terminology as subject headings or keywords, resulting in the identification of 101 additional records. The workflow is in Figure 1.

### Eligibility criteria

All clinical trials or retrospective studies that investigated ICIs in conjunction with antibiotics, probiotics, or FMT as study exposures were eligible for inclusion. The study population consisted of cancer patients receiving immunotherapy alone or in combination with other treatments. The following ICI regimens were considered eligible: anti-PD-1, anti-PD-L1, and anti-CTLA-4 agents, administered either as monotherapy or in combination. Included studies were required reporting a minimum of one efficacy outcome according to globally accepted oncologic assessment standards, including ORR, OS, or PFS.

### Selection of studies

For antibiotics and probiotics, eligible study designs included dual-arm prospective and retrospective cohort studies, whereas studies involving FMT included both single-arm and dual-arm designs. Included studies were required reporting therapeutic clinical effectiveness and patient outcomes observed during follow-up. Studies limited to alterations in the tumor immune microenvironment or microbiome sequencing alterations were excluded from the analysis. Studies in which the outcomes of interest were limited to adverse events, as well as reviews, protocols, case reports, editorials, and meta-analyses, were also excluded.

### Evaluation of risk of bias and methodological quality

Standardized risk of bias (ROB) assessment tools were applied to examine the methodological rigor of the included research. Different ROB assessment tools were used according to study design. ROB was assessed based on study type: randomized controlled trials (RCTs) were assessed using ROB 2.0^6^; non-RCTs were assessed using ROBINS-I tool.^7^ In addition, to comprehensively assess potential publication bias, Review Manager (version 5.4) was used to perform ROB analyses and summaries.

### Statistical methods and analysis

This study used Stata 18 (Stata Corp) and conducted the meta-analysis with the assistance of metafor (version 4.6.0), grid (version 4.4.1), meta (version 8.0.1), and forestplot (version 3.1.6) packages. ORR was designated as the primary endpoint, considered to be treatment response following ICIs combined with antibiotics, probiotics, or FMT. OS and PFS were evaluated as secondary endpoints to assess patient outcomes and long-term disease control. During data extraction and synthesis, baseline patient characteristics and study-level variables were systematically collected, including tumor type, ICI class, timing and route of administration, antibiotic class, probiotic type, FMT source, study design, and therapy line. For the single-arm analyses of FMT, ORR along with its 95% CI and *P* value were extracted. For dual-arm studies of antibiotics and probiotics, odds ratios (ORs) were calculated for ORR, whereas hazard ratios (HRs) were to assess survival outcome, together with their corresponding 95% CIs being estimated. To comprehensively evaluate the impact of 3 intervention-related factors on ICI efficacy as well as patient endpoints, we used fixed-effects or random-effects models. Statistical significance was defined as *P* < .05. Between-group comparisons of median OS (mOS) and median PFS (mPFS) were performed for antibiotic- and probiotic-related studies.

## Results

This meta-analysis included 3 categories of studies, comprising a total of 106 studies and 77 210 patients: 76 studies on antibiotics involving 71 779 patients,^8–83^ 15 studies on probiotics involving 5038 patients,^3^^,^^84–97^ and 15 studies on FMT involving 393 patients.^98–112^

### Association of antibiotics with ICI efficacy and survival

#### Study characteristics of antibiotic cohort

Most studies incorporated in this meta-analysis on antibiotics adopted a retrospective cohort design, with publication periods mainly spanning from 2015 to 2025 and covering multiple regions across Europe, North America, and Asia. The study populations primarily include patients with advanced-stage solid malignancies, among whom non–small cell lung cancer (NSCLC) was largest, while other tumor types included melanoma, RCC, hepatocellular carcinoma (HCC), and urothelial carcinoma (UC). Antibiotic exposure was generally defined as systemic use within a specified period preceding, with the most commonly applied window ranging from 30-60 days before ICI initiation to 30-42 days following initiation. The antibiotics used were mainly broad-spectrum agents, including β-lactams, fluoroquinolones, and cephalosporins, with routes of administration including both oral and intravenous delivery (Table S4).

#### Association of antibiotic exposure with ICI efficacy

**Figure 2.**
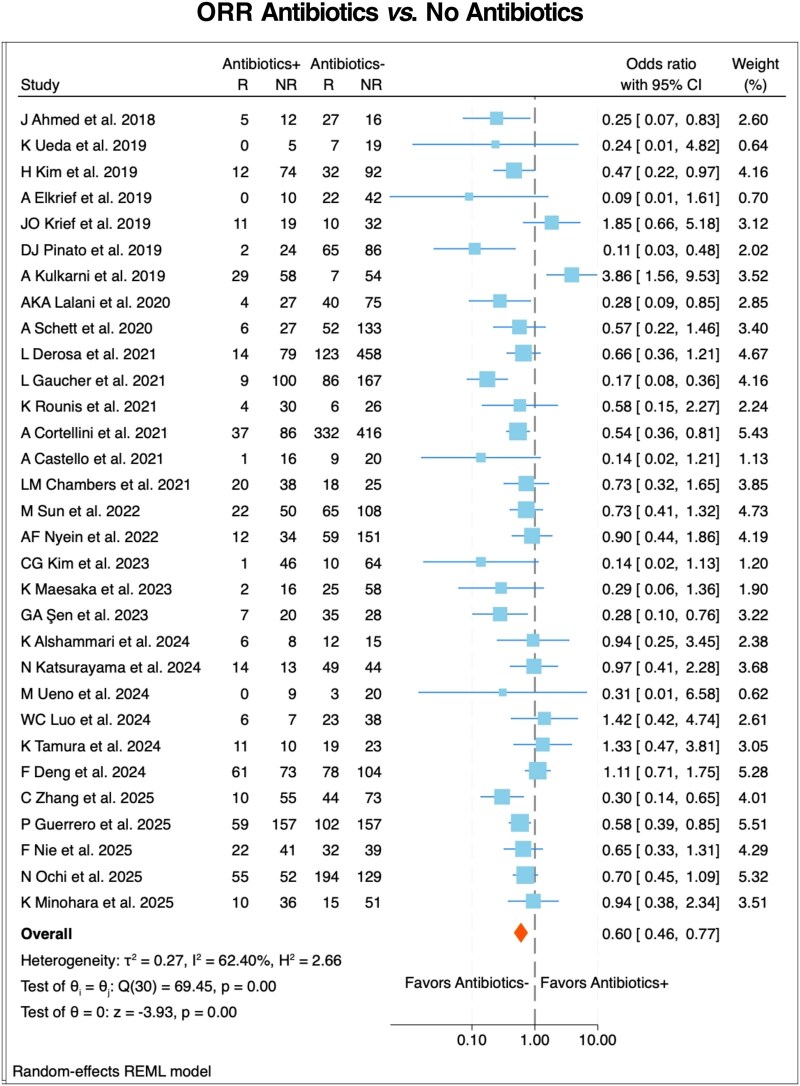
Meta-analysis of ORR in antibiotics cohort. Multivariable OR estimates were used. Studies are presented in temporal order of publication; a larger square indicates a larger study weight. The horizontal lines represent the 95% CI, and the diamond represents the pooled effect estimate. NR, nonresponse; ORR, objective response rate; R, response.

Compared with patients who were not exposed to antibiotics, those exposed to antibiotics had significantly reduced probability of achieving an ORR (OR = 0.60, 95% CI, 0.46-0.77, *P* < .001) suggesting that antibiotic-associated alterations of intestinal microbial flora attenuate effectiveness of immunotherapy. Between-study heterogeneity was substantial (*I*^2^= 62.4%, Figure 2). We then conducted subgroup analyses. In tumor type-specific subgroups, use of antibiotics was linked to decreased ORR in most solid tumors, including NSCLC, melanoma, biliary tract cancer. In NSCLC, the random-effects model suggested a significant association linking antibiotics to decreased ORR (OR = 0.76, 95% CI, 0.59-0.98, *P* = .03). When stratified by line of therapy, antibiotic use also showed an unfavorable trend among patients receiving first-line therapy, mixed line therapy under the random-effects model. In subgroup analyses according to ICI regimens, a clear association between antibiotic exposure and decreased ORR was observed under PD-1-based monotherapy and combination of PD-1, PD-L1, and CTLA-4 inhibitors. We further stratified by trial type. Retrospective studies demonstrated a significant ORR (OR = 0.61, 95% CI, 0.46-0.80, *P* < .001) (Table S5).

#### Association of antibiotic exposure with survival outcome

**Figure 3.**
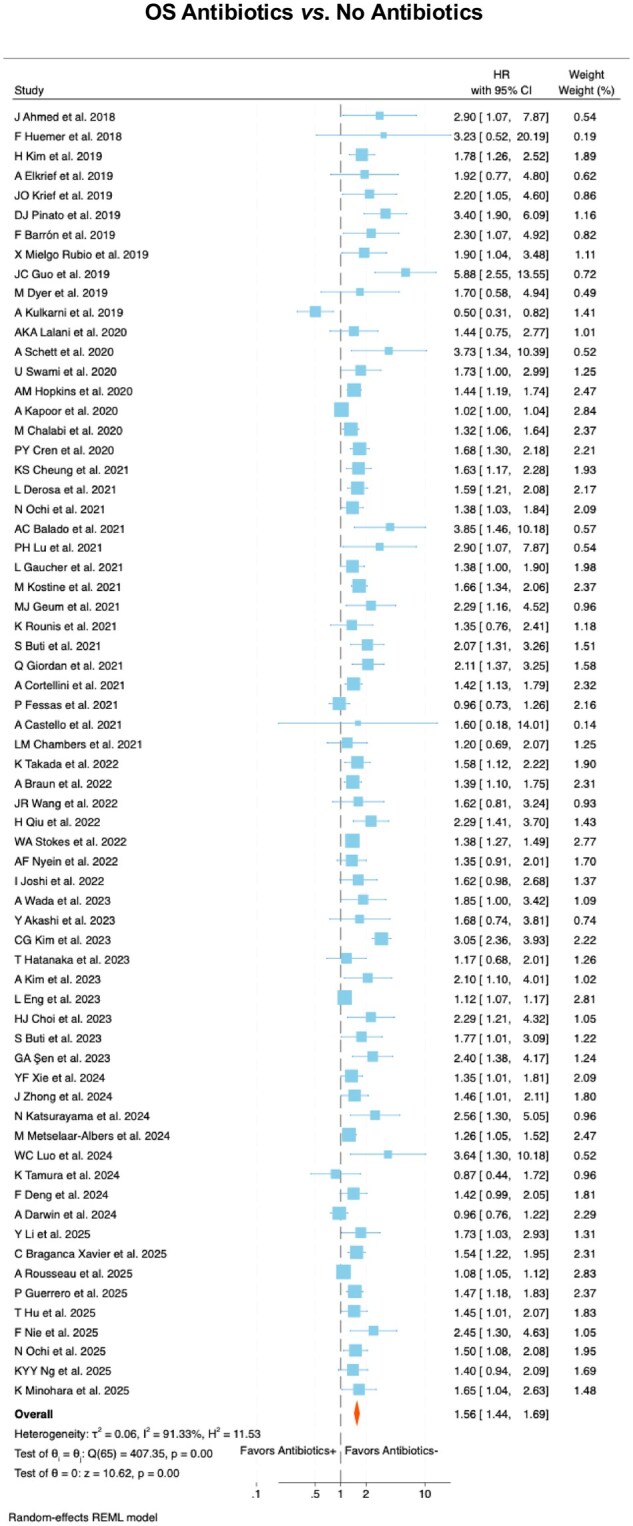
Meta-analysis of OS in antibiotics cohort. Multivariable HR estimates were used. Studies are presented in temporal order of publication; a larger square indicates a larger study weight. The horizontal lines represent the 95% CI, and the diamond represents the pooled effect estimate. HR, hazard ratio; OS, overall survival.

**Figure 4.**
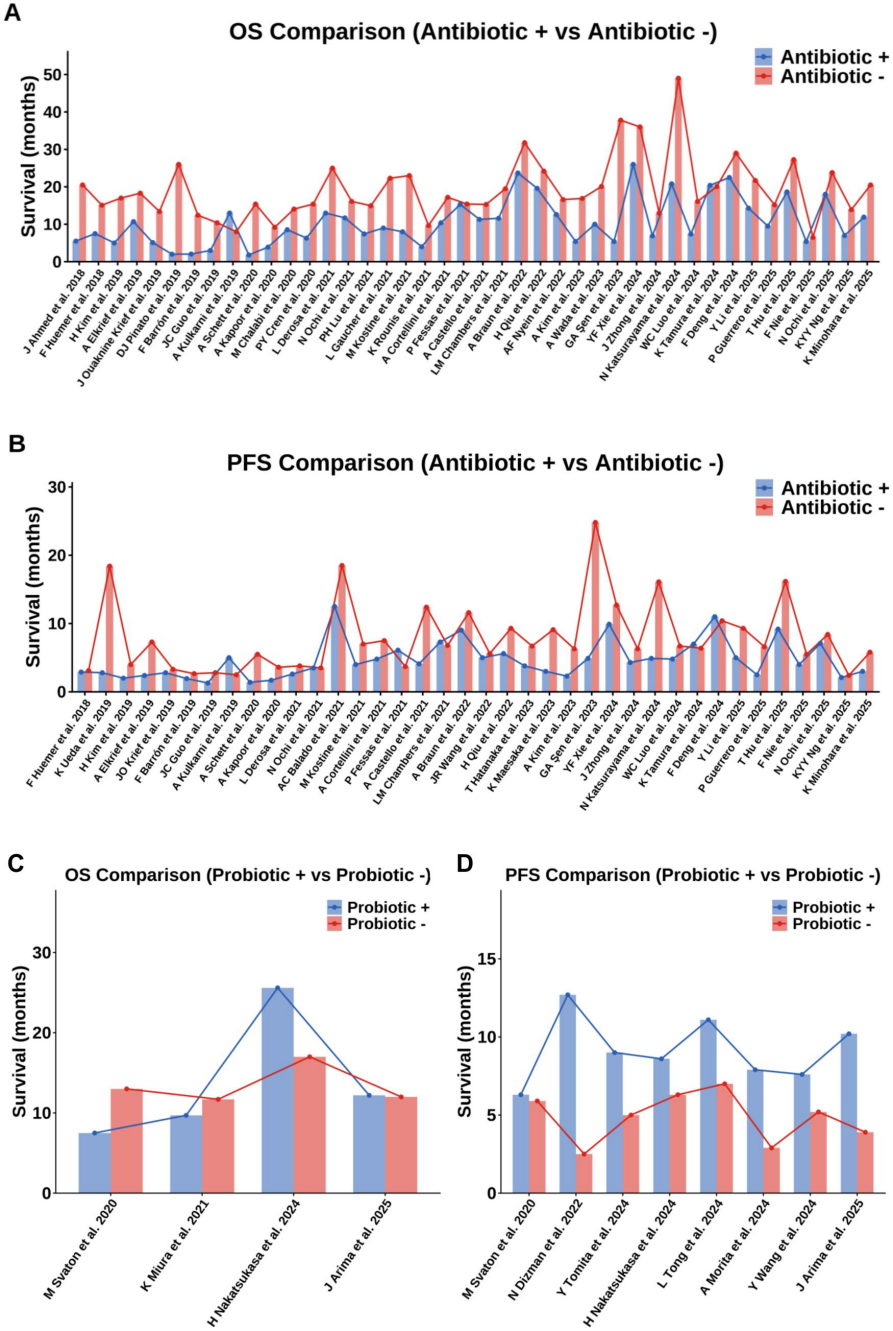
Comparison of mOS and mPFS between antibiotics^+^, antibiotics− patients and probiotic^+^, probiotic− treated with ICI. This figure summarizes the reported mOS and mPFS from individual studies comparing antibiotics^+^ with antibiotics^-^ (A, B) and probiotic^+^ with probiotic^-^ (C, D) in the context of ICI therapy. Each pair of bars corresponds to a single study, ordered chronologically by publication year. Overall, most studies show shorter mOS and mPFS in the antibiotics^+^ group and longer mOS and mPFS in the probiotic^+^ group, illustrating a consistent trend toward inferior survival associated with antibiotics exposure and superior survival associated with probiotics exposure. These descriptive findings complement the HR-based meta-analysis and provide an intuitive visualization of survival differences across studies. HR, hazard ratio; OS, overall survival; PFS, progression free survival.

**Figure 5.**
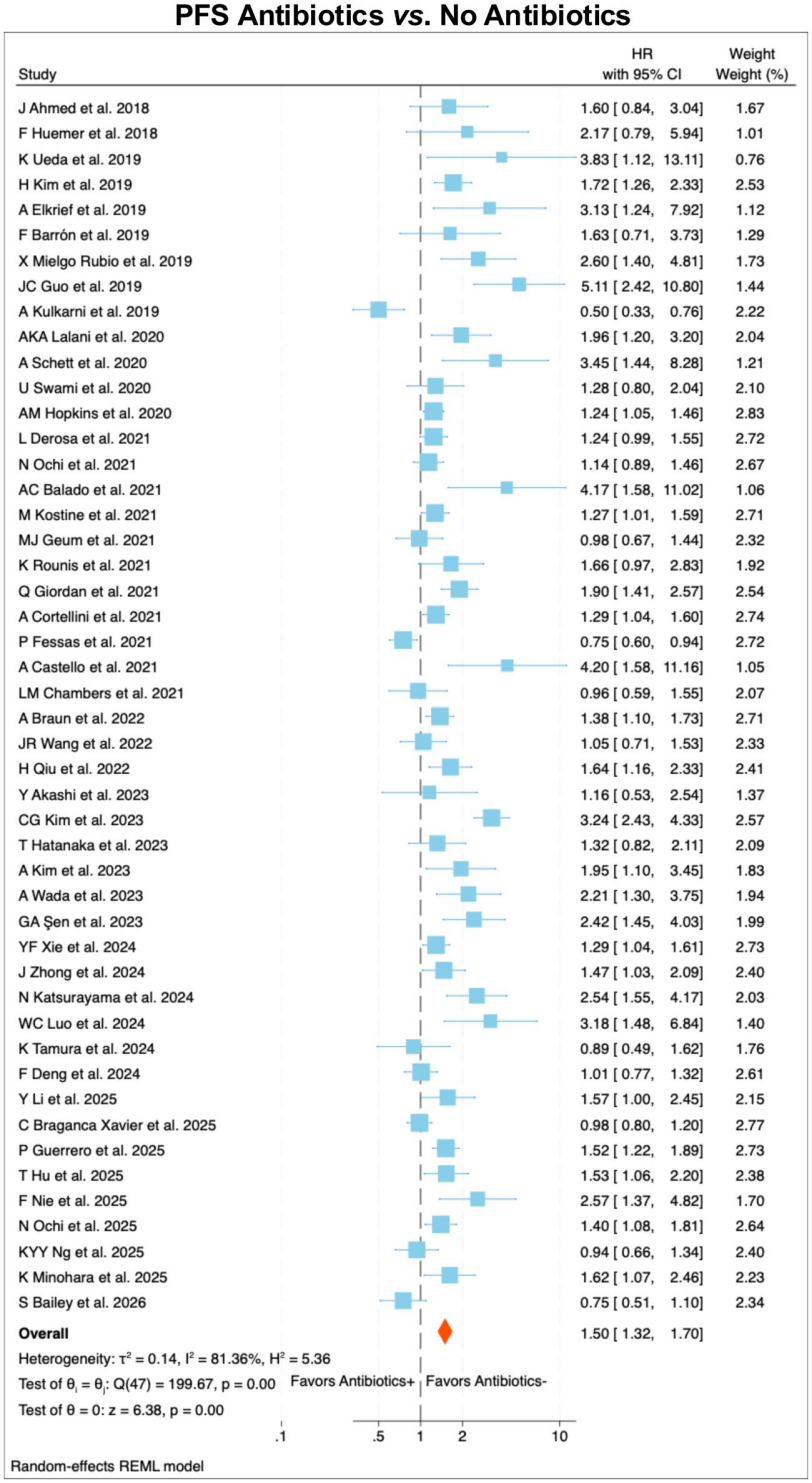
Meta-analysis of PFS in antibiotics cohort. Multivariable HR estimates were used. Studies are presented in temporal order of publication; a larger square indicates a larger study weight. The horizontal lines represent the 95% CI, and the diamond represents the pooled effect estimate. HR, hazard ratio; PFS, progression free survival.

Random-effects model showed that antibiotics were significantly correlated with shortened OS (HR = 1.56., 95% CI, 1.44-1.69, *P* < .001, Figures 3 and 4A). Substantial between-study heterogeneity was observed (*I*^2^= 91.33%), indicating differences in patient characteristics and patterns of antibiotic exposure across studies (Figure 3). Besides, patients with antibiotic exposure had shorter PFS (HR = 1.50, 95% CI, 1.32-1.70, *P* < .001, Figures 4B and 5) with high heterogeneity (*I*^2^= 81.36%, Figure 5). When stratified by study design, both retrospective and prospective studies showed stable and significant detrimental effects of antibiotics on OS and PFS (Tables S6 and S7). When stratified according to ICI regimens, subgroup analyses revealed that antibiotics significantly shortened OS within all kinds of regimens (Table S6). Shortened PFS was also observed in patients receiving various ICIs who were exposed to antibiotics, except for anti-PD-1 with anti-CTLA-4 therapy and experimental immunotherapy (Table S7). Subgroup analyses by therapy line showed that antibiotics were linked to unfavorable outcomes for OS and PFS in different treatment settings (Table S6 and S7). In tumor type-specific subgroup analyses for OS, a significant elevation in risk of death was linked to antibiotic use in all cancer types except for gynecologic malignancies (Table S6). A similarly unfavorable trend was observed for PFS in esophageal squamous cell carcinoma, gastric cancer (GC), head and neck cancer (HNC), NSCLC, RCC, SCLC, and UC (Table S7).

#### Leave-one-out sensitivity analyses and funnel plots in antibiotic cohort

Leave-one-out sensitivity analyses conducted for ORR, OS, and PFS yielded consistent pooled estimates with minimal variation, indicating that no individual study exerted a decisive influence on the overall conclusions **(**Figures S1-S3, see online supplementary material for a color version of these figures). Funnel plots were generated to assess potential publication bias for ORR, OS, and PFS in the antibiotic^+^ versus antibiotic^-^ analysis (Figure S4A-C, see online supplementary material for a color version of this figure). Funnel plots for ORR and PFS were generally acceptable, although some dispersion and asymmetry of study estimates remained, indicating that between-study heterogeneity and potential small-study effects could not be fully excluded. The OS funnel plot showed more evident asymmetry and a less stable distribution, suggesting that the OS model should be interpreted with particular caution. Therefore, the findings related to antibiotic use, especially those for OS, require further validation in larger, higher-quality studies.

### Association of probiotic with ICI efficacy and survival

#### Study characteristics of probiotic cohort

The included probiotic-related studies were composed of both retrospective cohort investigations and prospective early-phase clinical trials. The study regions mainly included Japan (7), the United States (3), and China (3). The study populations largely consisted of patients with advanced solid tumors receiving ICI therapy, among whom NSCLC (8) was the most common. Common probiotic formulations included CBM588 (6), *Bifidobacterium* species (1), SER-401 (1), *Lacticaselbacillus paracasei* (1), and compound probiotic preparations (3). Immunotherapy regimens were based on PD-1, PD-L1, and CTLA-4 inhibitors (Table S8).

#### Association of probiotic administration with ICI efficacy

**Figure 6.**
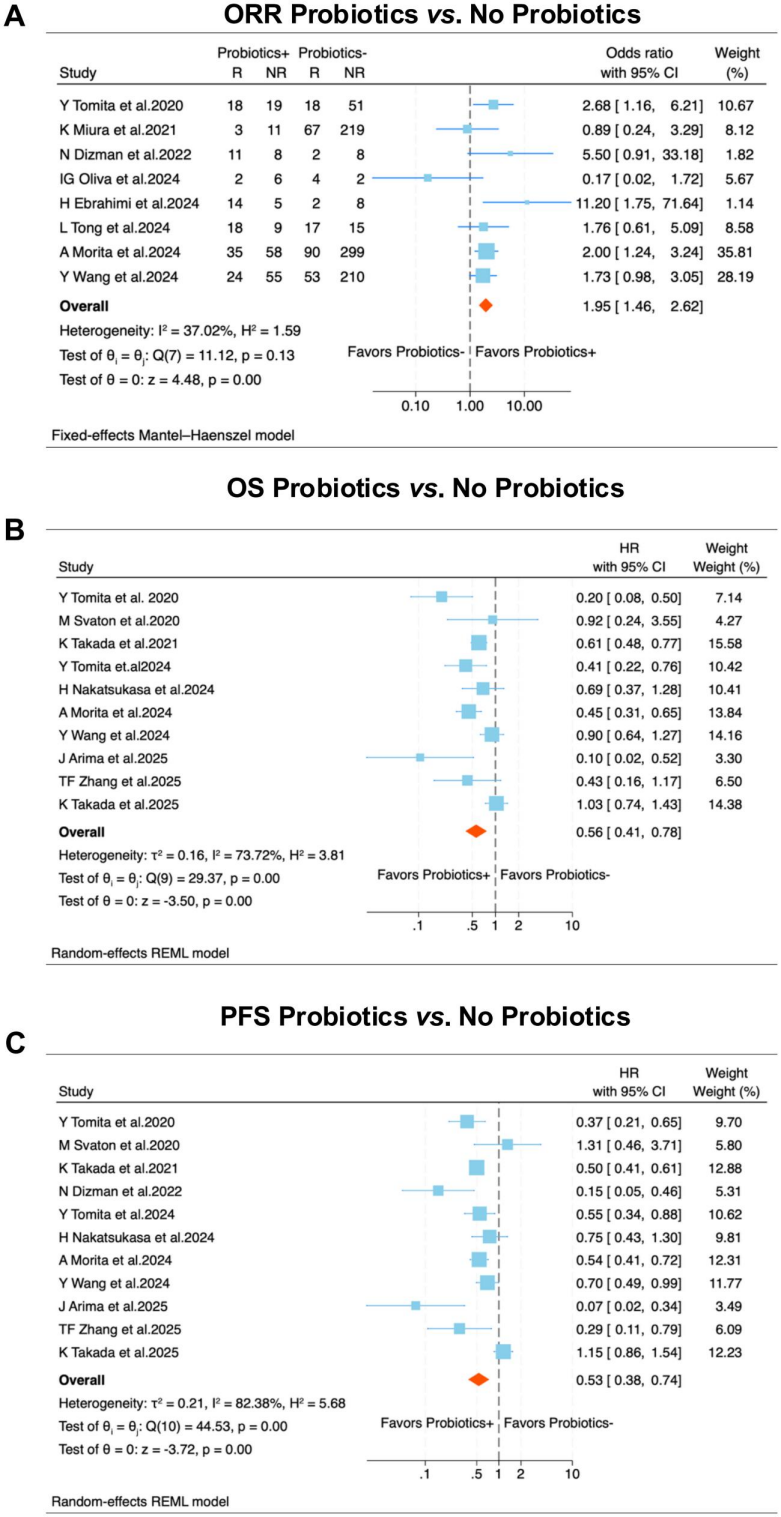
Meta-analysis of (A) ORR, (B) OS, and (C) PFS in probiotic cohort. Multivariable OR (A) and HR (B, C) estimates were used. Studies are presented in temporal order of publication; a larger square indicates a larger study weight. The horizontal lines represent the 95% CI, and the diamond represents the pooled effect estimate. HR, hazard ratio; NR, nonresponse; ORR, objective response rate; OS, overall survival; PFS, progression-free survival; R, response.

There is a statistically meaningful relationship between probiotic use and increased ORR (OR = 1.95, 95% CI, 1.46- 2.62, *P* < .001). Low degree of heterogeneity was detected among studies (*I*^2^= 37.02%), indicating relatively minor differences in clinical characteristics and probiotic intervention strategies across studies (Figure 6A). Probiotic use was associated with increased ORR in certain subgroups. Both retrospective studies and prospective studies demonstrated a stable and significant positive association with ORR (Table S9). Anti-PD-1 monotherapy and combined with anti-PD-L1 treatment both reported a statistically significant effect, suggesting that probiotics may exert great effects in diverse immunotherapy (Table S9). Subgroup analyses by therapy line showed that probiotics were associated with higher ORR in different treatment settings (Table S9). Both NSCLC and RCC subgroups demonstrated a significant increase in ORR, suggesting a significant association between probiotic use and higher ORR (Table S9). However, as several findings were based on a single study, further evidence is required for confirmation.

#### Association of probiotic administration with survival outcome

Probiotic use was significantly correlated with improved OS (HR = 0.56, 95% CI, 0.41-0.78, *P* < .001, Figures 4C and Figure 6B) and PFS (HR = 0.53, 95% CI, 0.38-0.74, *P* < .001, Figures 4D and Figure 6C). When stratified by study design, retrospective studies demonstrated significant improvements in OS and PFS (Tables S10 and S11). Subgroup analyses by immunotherapy regimen indicated that in all immunotherapy regimens except for PD-L1, probiotic use significantly improved OS and PFS (Tables S10 and S11). Subgroup analyses by therapy line showed that probiotic use was related to meaningful improvements in OS and PFS in ≥ second-line therapy (Tables S10 and Table S11), indicating consistent and significant benefits in survival outcomes in advanced cancer patients. Subgroup analysis by tumor type indicated that probiotics markedly improved OS in NSCLC and UC (Table S10); probiotics also improved PFS in all subgroups of cancer types (Table S11).

#### Leave-one-out sensitivity analyses and funnel plots in probiotic cohort

Leave-one-out sensitivity analyses conducted for ORR, OS, and PFS yielded consistent pooled estimates with minimal variation, indicating that no individual study exerted a decisive influence on the overall conclusions (Figure S5-S7, see online supplementary material for a color version of these figures). Funnel plots showed no obvious marked asymmetry for OS and PFS, while the ORR funnel plot showed mild asymmetry, possibly due to the limited number of included studies. Although no strong evidence of substantial publication bias was observed, these findings should be interpreted with caution especially ORR because of the relatively small number of studies included in each analysis and further validation in larger, high-quality studies are required (Figure S8, see online supplementary material for a color version of this figure).

### Association of FMT with ICI efficacy and survival

#### Study characteristics of FMT cohort

FMT-related studies were mainly reported during the period from 2021 to 2026, and 6 of them were conducted in China. The study designs were predominantly phase 1/2 clinical trials or exploratory studies, with only a limited number adopting randomized controlled designs (1). The included patient populations primarily consisted of individuals with advanced solid tumors, including RCC (2), melanoma (4), NSCLC (2), CRC (1), GC (1), and other solid malignancies. One included study was treated as two independent cohorts because it analyzed two cancer types separately.^110^ Therapy lines of most studies are mainly at second-line or later (9). The common route of administration was oral delivery FMT capsules (11) and colonoscopy (6). Immunotherapy regimens primarily consisted of PD-1 inhibitor monotherapy (12) (Table S12).

#### Association of FMT administration with ICI efficacy

**Figure 7.**
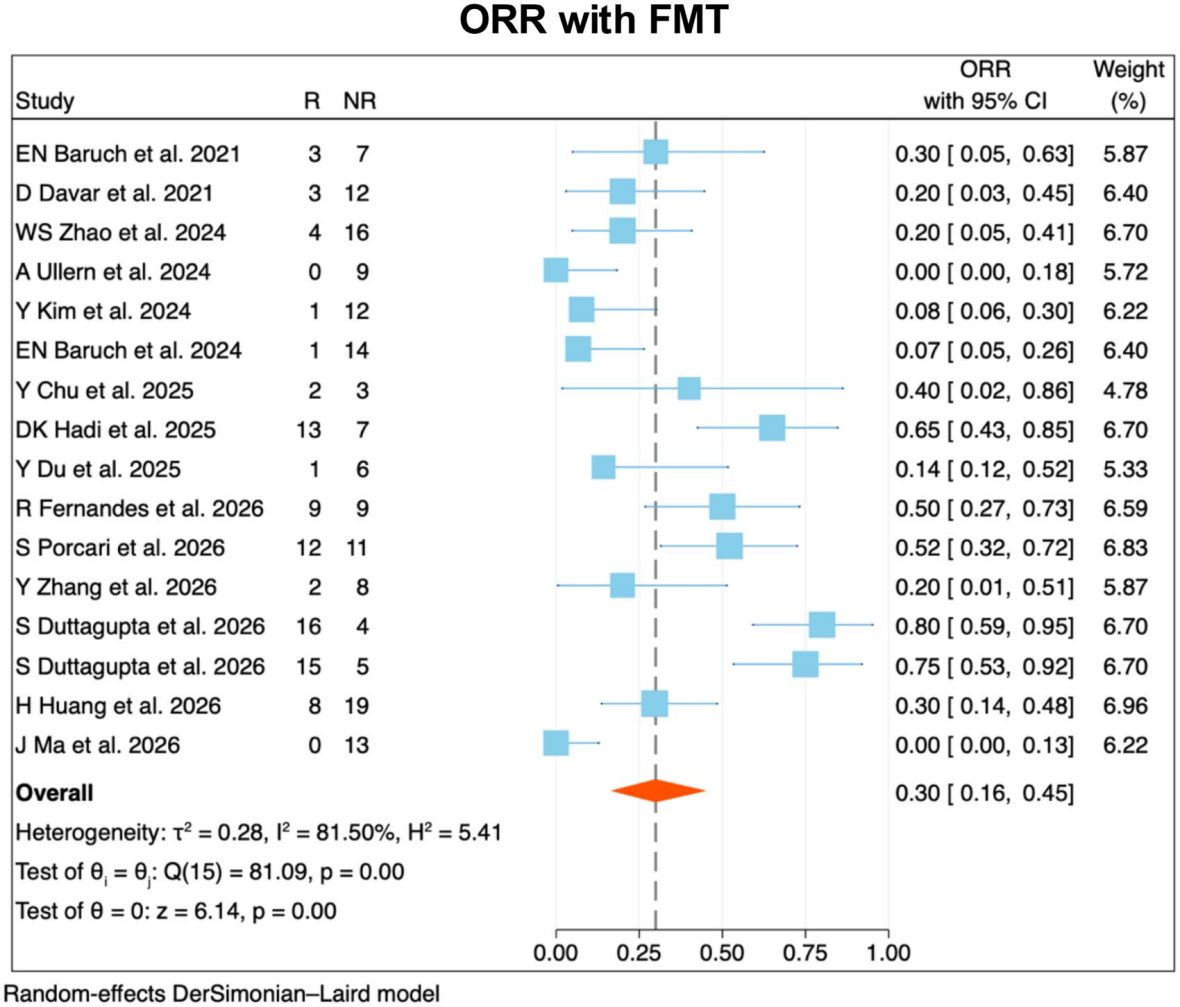
Meta-analysis of ORR in FMT cohort. Response events and total sample sizes were used to estimate pooled proportions with corresponding 95% CIs. Studies are presented in temporal order of publication; a larger square indicates a larger study weight. The horizontal lines represent the 95% CI, and the diamond represents the pooled effect estimate. NR, nonresponse; ORR, objective response rate; R, response.

The pooled estimate derived from a random-effects model indicated that FMT combined with ICIs achieved a moderate level of ORR in the overall population (ORR = 0.30, 95% CI, 0.16-0.45, *P* < .001, Figure 7). Stratification by study design revealed that both single-arm and dual-arm studies reported statistically significant ORRs for FMT combined with ICIs, indicating a degree of robustness across different study designs (Table S13). And higher ORR can be seen across different immunotherapy regimens (Table S13). Stratified analyses by therapy line showed that FMT exhibited clinical efficacy in patients treated in earlier lines rather than in later lines (Table S13). Subgroup analyses of tumor types demonstrated high ORRs of various cancer types except for HCC (Table S13). Analyses by donor source showed ORR levels of FMT from healthy donors are higher than those of responder-derived donors, although high heterogeneity was observed in both subgroups (Table S13). With respect to administration routes, oral FMT capsules yielded a relatively better ORR than colonoscopic administration; however, the findings were primarily based on a small number of studies and therefore require further validation (Table S13).

#### Leave-one-out sensitivity analyses and funnel plots in FMT cohort

Sequential leave-one-out sensitivity analyses indicated that the pooled ORR remained generally consistent after exclusion of any single study (Figure S9, see online supplementary material for a color version of this figure), funnel plot inspection revealed mild asymmetry, indicating that potential publication bias or small-study effects could not be completely excluded. Nevertheless, because the included FMT studies were limited in number and generally had small sample sizes with considerable clinical heterogeneity, these findings should be interpreted cautiously (Figure S10, see online supplementary material for a color version of this figure).

### Risk of bias

The majority of studies were evaluated as having a low ROB in outcome assessment, completeness of follow-up, and completeness of outcome reporting, suggesting that the ascertainment of the endpoints (ORR, OS, and PFS) were relatively reliable. However, because the included studies were predominantly retrospective observational cohorts, inherent limitations were present with respect to the randomness of exposure allocation and the control of confounding factors, rendering selection bias and confounding bias unavoidable in some studies (Figures S11 and S12, see online supplementary material for a color version of these figures). In the randomization process and allocation concealment of RCT studies, there also existed selection bias and confounding bias (Figure S13, see online supplementary material for a color version of this figure).

## Discussion

Gut microecology profoundly exerts a substantial impact on the efficacy and tolerability of ICIs through its role in modulating host immune function and the tumor microenvironment. Recent comprehensive reviews have highlighted that gut microorganisms play a regulatory role in CD8^+^ T-cell function, dendritic cell maturation, and the maintenance of regulatory T-cell balance, thereby orchestrating antitumor immune responses.^1^ Our study systematically synthesized the effects of 3 gut microbiota-related factors—antibiotics, probiotics, and FMT—with respect to ICIs. Overall, our meta-analysis indicates that gut microbiota has the potential to improve the clinical effectiveness of ICIs and patients’ outcomes.

With respect to antibiotic exposure, this study demonstrated that antibiotics were related to lower ORR, shorter OS, and reduced PFS. These findings are consistent with recent literature suggesting that antibiotic-induced dysbiosis compromises intestinal and systemic immune surveillance and antitumor immune activity. As a highly plastic environmental factor, disruption of the gut microbiota not only attenuates treatment responses but also adversely affects patient survival outcomes. Moreover, antibiotic-associated reductions in microbial diversity have been associated with reduced immunotherapy effectiveness across multiple cancer types, underscoring the clinical importance of judicious antimicrobial stewardship during immunotherapy.

In contrast, probiotics—representing a relatively mild form of microbiota modulation—showed potential associations with improved treatment efficacy and survival outcomes in this analysis. Recent studies suggest that probiotics and related microbiota-supportive strategies, including prebiotics and postbiotics, may not only reshape gut microbial composition but also activate antitumor immune responses through specific microbial metabolites, thereby enhancing ICI responsiveness.^113^ However, current probiotic studies exhibit substantial heterogeneity in terms of strain selection, applicable cancer types, and compatibility with immunotherapy regimens, leading to inconsistent results across studies. Consequently, a more promising future direction may lie in “precision probiotics” or synthetic microbial consortia, which stratify patients based on microbiota features and immune phenotypes and elucidate therapeutic heterogeneity through integrated multi-omics and immunologic readouts.^114^

Our meta-analysis indicates that FMT may enhance antitumor immune responses and potentially overcome immunotherapy resistance in selected patient populations. More aggressive microbiota remodeling strategies, particularly FMT, have shown frontline potential for overcoming immunotherapy resistance in both experimental and early clinical settings. Emerging clinical observations and small-scale studies indicate that combining FMT with ICIs may reverse resistance to ICIs in certain individuals by facilitating beneficial microbial engraftment and immune system reprogramming. With the development of safer and more convenient approaches—such as capsule-based FMT—the clinical feasibility and translational potential of FMT are gradually being validated.^115^ Nevertheless, substantial challenges remain, including donor selection and standardization, precise microbiota matching, and long-term safety assessment, all of which may influence the consistency and durability of therapeutic effects.

There exist limitations: First, a key limitation of this review is that most included studies were retrospective cohort analyses, which inevitably introduces potential selection bias, information bias, and residual confounding. As a result, the associations observed between microbiome-related factors and immunotherapy outcomes should be interpreted cautiously. Prospective, well-controlled studies are needed to validate these findings and better clarify causality. Second, substantial heterogeneity was observed across included studies. Third, the possibility of publication bias and small-sample effects cannot be fully excluded, particularly for probiotic and FMT analyses, given the relatively few studies and the predominance of early exploratory trials. Fourth, the detail characteristics of patients were not consistently reported.

In conclusion, this meta-analysis provides robust evidence that antibiotic-induced disruption of gut microbiota is associated with an attenuation of the therapeutic efficacy of ICIs. However, the use of probiotics and FMT can enhance the immune efficacy. These findings highlight the critical role of the gut microbiome as a key modulator of host immunity and treatment response in patients receiving ICIs.

## Supplementary Material

oyag264_Supplementary_Data

## Data Availability

The datasets supporting the conclusions of this article are included within the article and its additional files.
