# Supplementary material for: The use of antibiotic, probiotic, and fecal microbiota transplantation in modulating immunotherapy efficacy and survival: a systematic review and meta-analysis of clinical outcomes
Source: Oncologist. 2026 Jul 10;31(8):oyag264. doi: 10.1093/oncolo/oyag264 (PMC13401492; doi:10.1093/oncolo/oyag264)
Supplement: oyag264_Supplementary_Data [file oyag264_supplementary_data.zip › 21-Jul-2026_023449_Supplementary_Data.zip!/supplement_figure.pdf]

Figure S1 Sensitivity analysis of ORR: Antibiotics *vs*. No Antibiotics

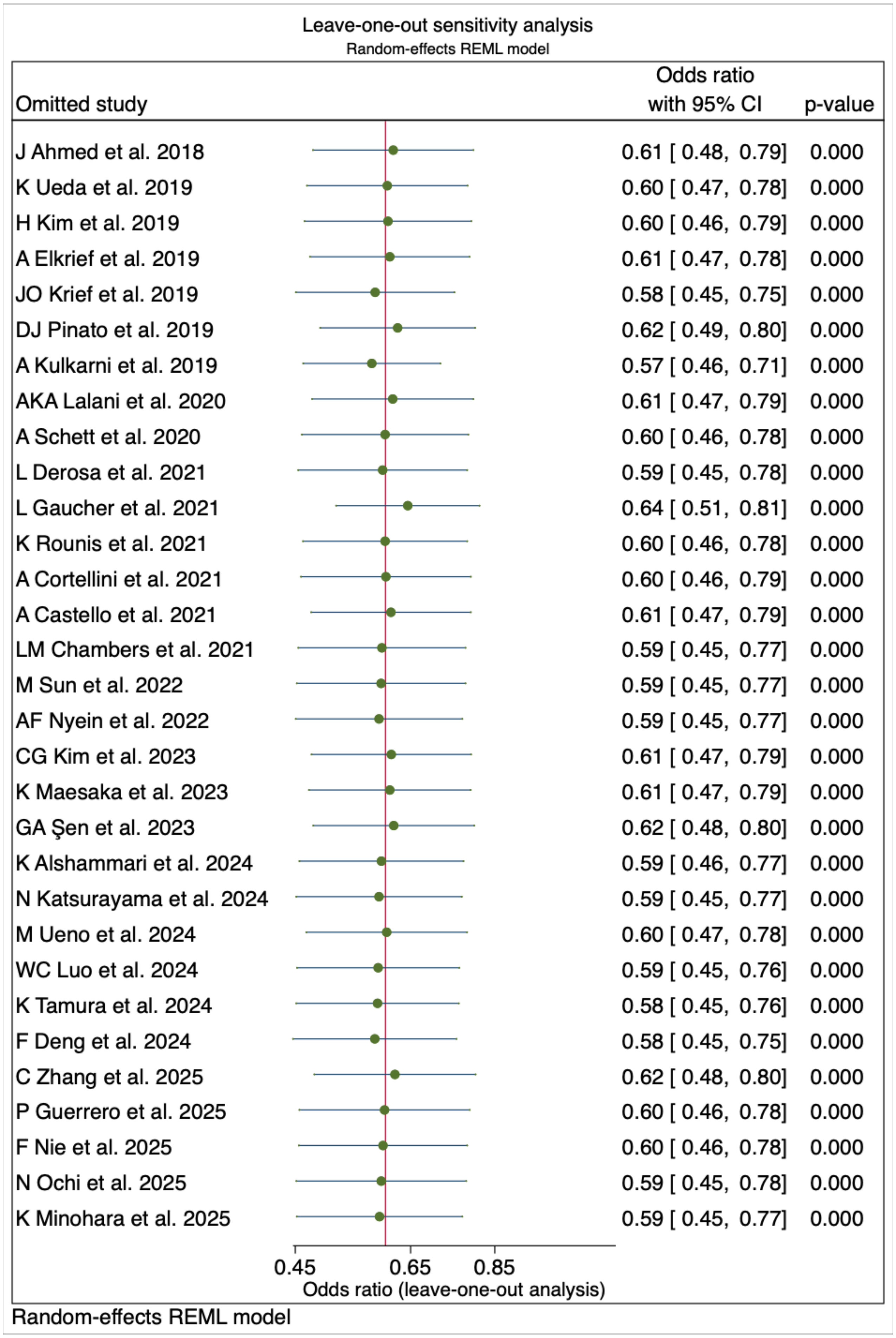

Figure S1. Leave-one-out sensitivity analysis of ORR in patients receiving Antibiotic using a random-effects model (REML). ORR, objective response rate; CI, confidence interval.

Figure S2 Sensitivity analysis of OS: Antibiotics *vs*. No Antibiotics

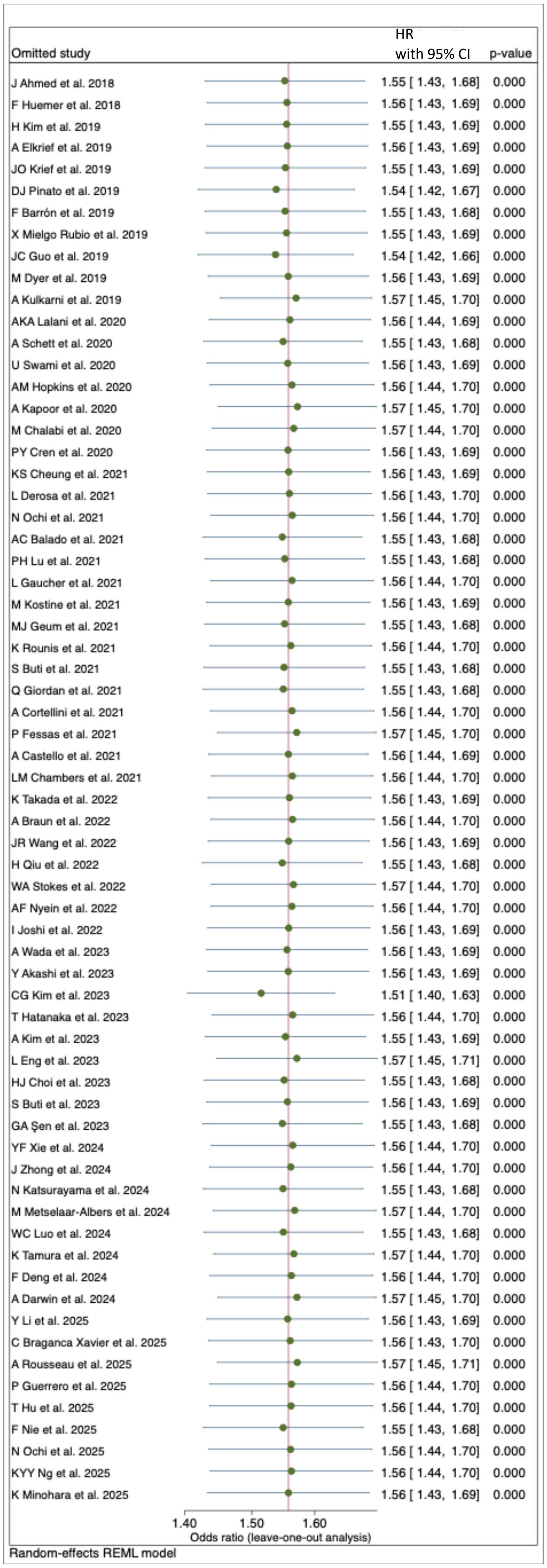

Figure S2. Leave-one-out sensitivity analysis of OS in patients receiving Antibiotic using a random-effects model (REML). HR, hazard ratio; CI, confidence interval

Figure S3 Sensitivity analysis of PFS: Antibiotics *vs*. No Antibiotics

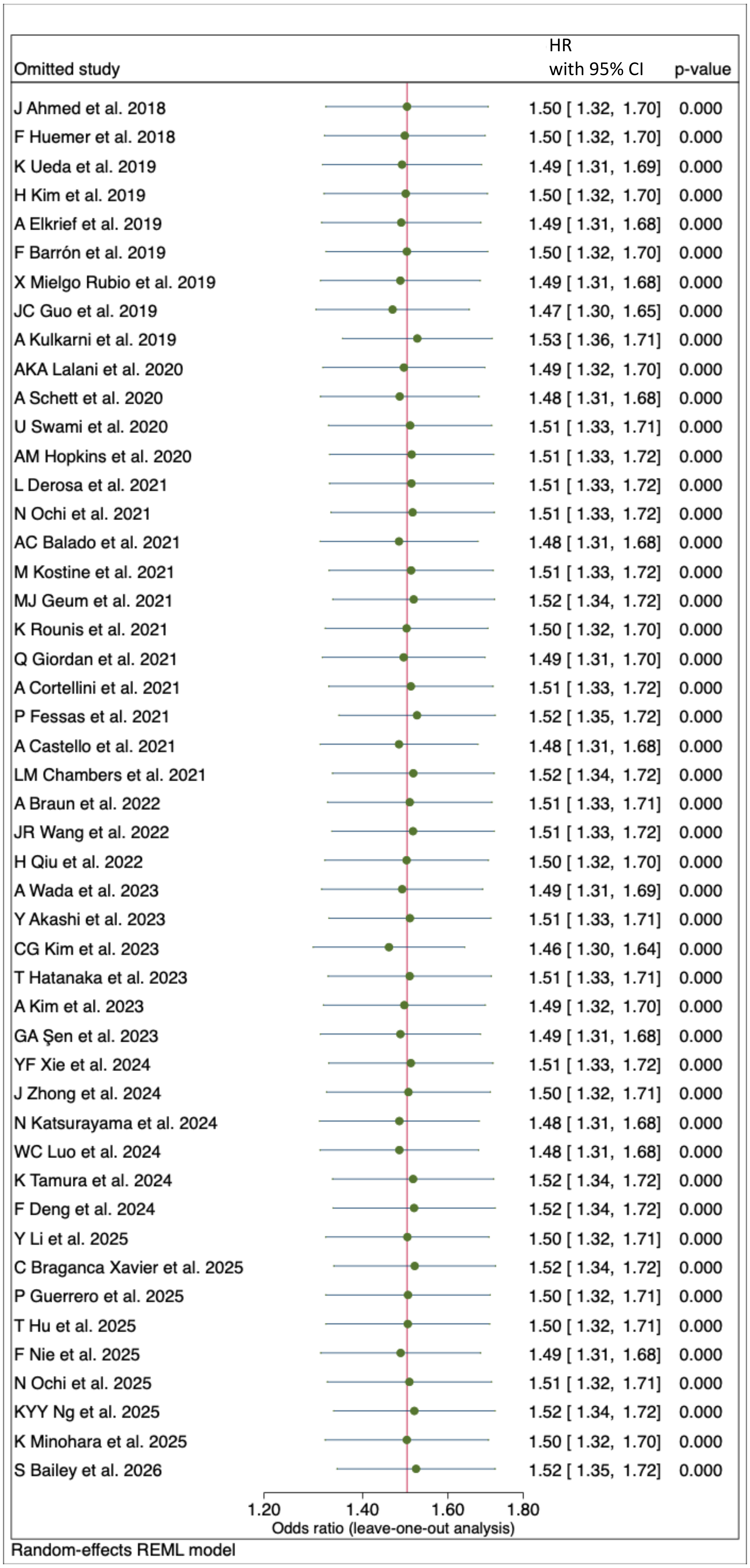

Figure S3. Leave-one-out sensitivity analysis of PFS in patients receiving Antibiotic using a random-effects model (REML). HR, hazard ratio; CI, confidence interval

Figure S4 Funnel plot of ORR, OS and PFS: Antibiotics *vs*. No Antibiotics

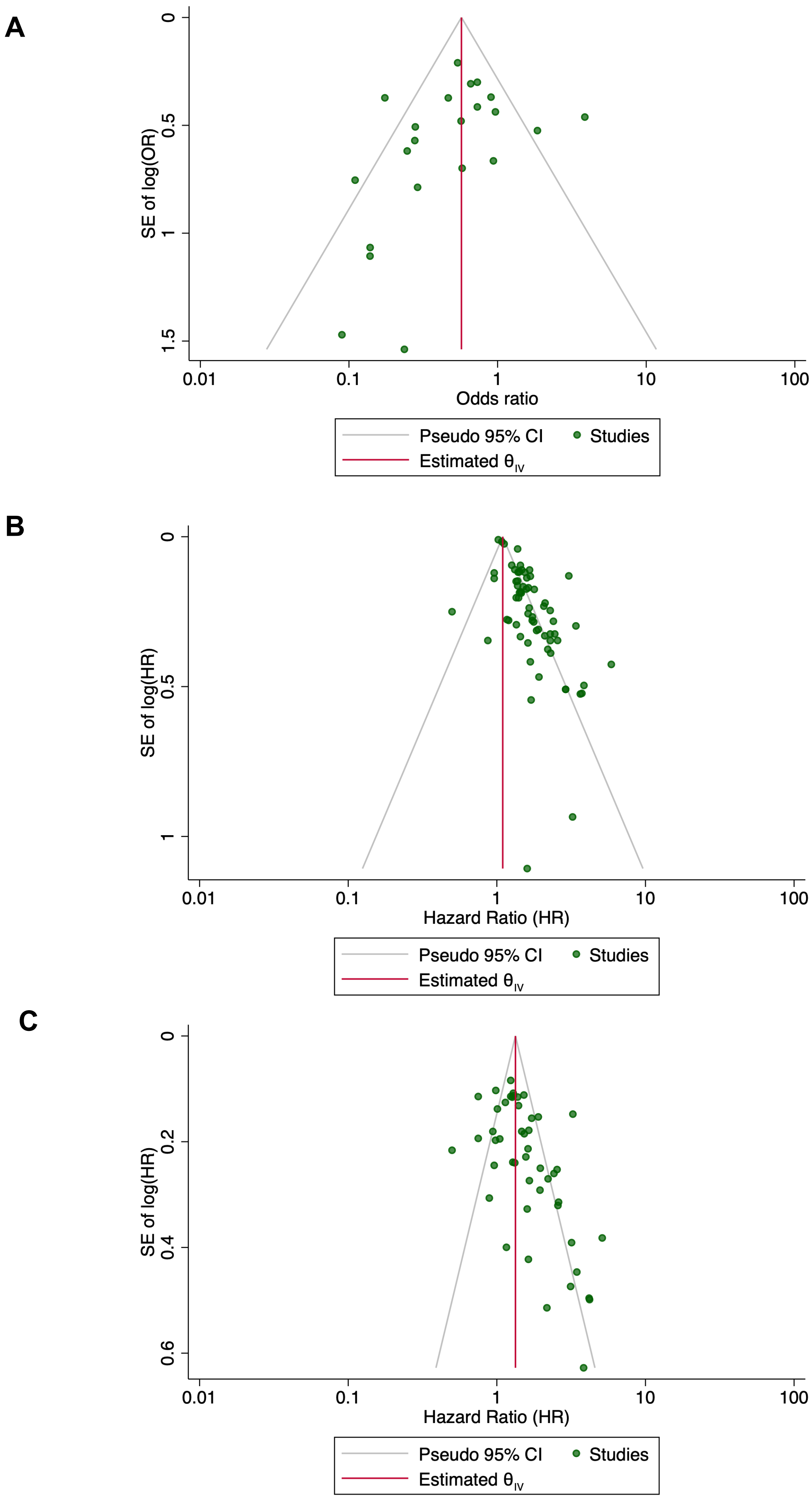

Figure S4. Funnel plot assessing publication bias for ORR **(A)**, OS **(B)** and PFS **(C)** in patients receiving antibiotics. ORR, objective response rate; OR, odds ratio; OS, overall survival; HR, hazard ratio, PFS, progression-free survival

Figure S5 Sensitivity analysis of ORR: Probiotics *vs*. No Probiotics

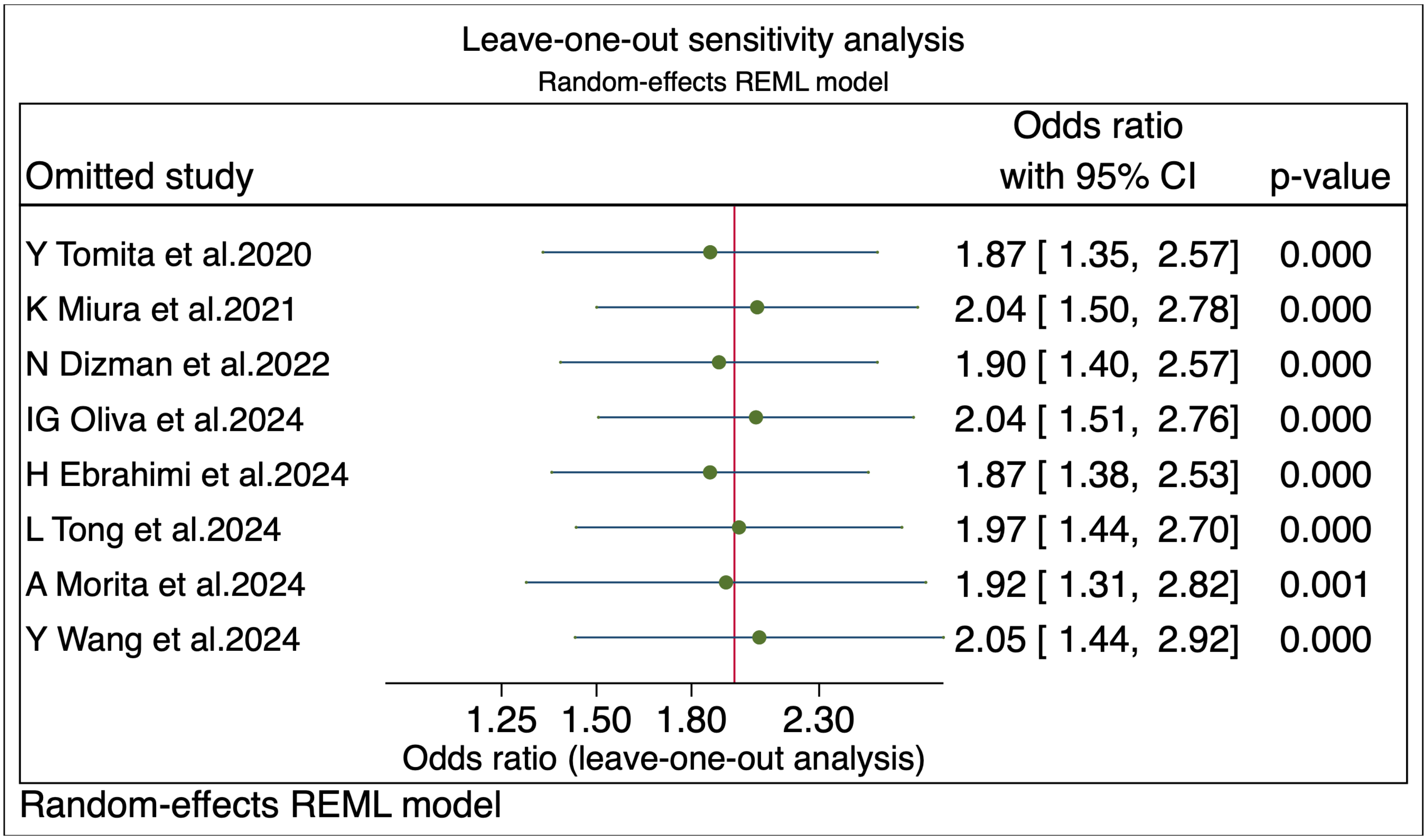

Figure S5. Leave-one-out sensitivity analysis of ORR in patients receiving Probiotic using a random-effects model (REML). ORR, objective response rate; CI, confidence interval.

Figure S6 Sensitivity analysis of OS: Probiotics *vs*. No Probiotics

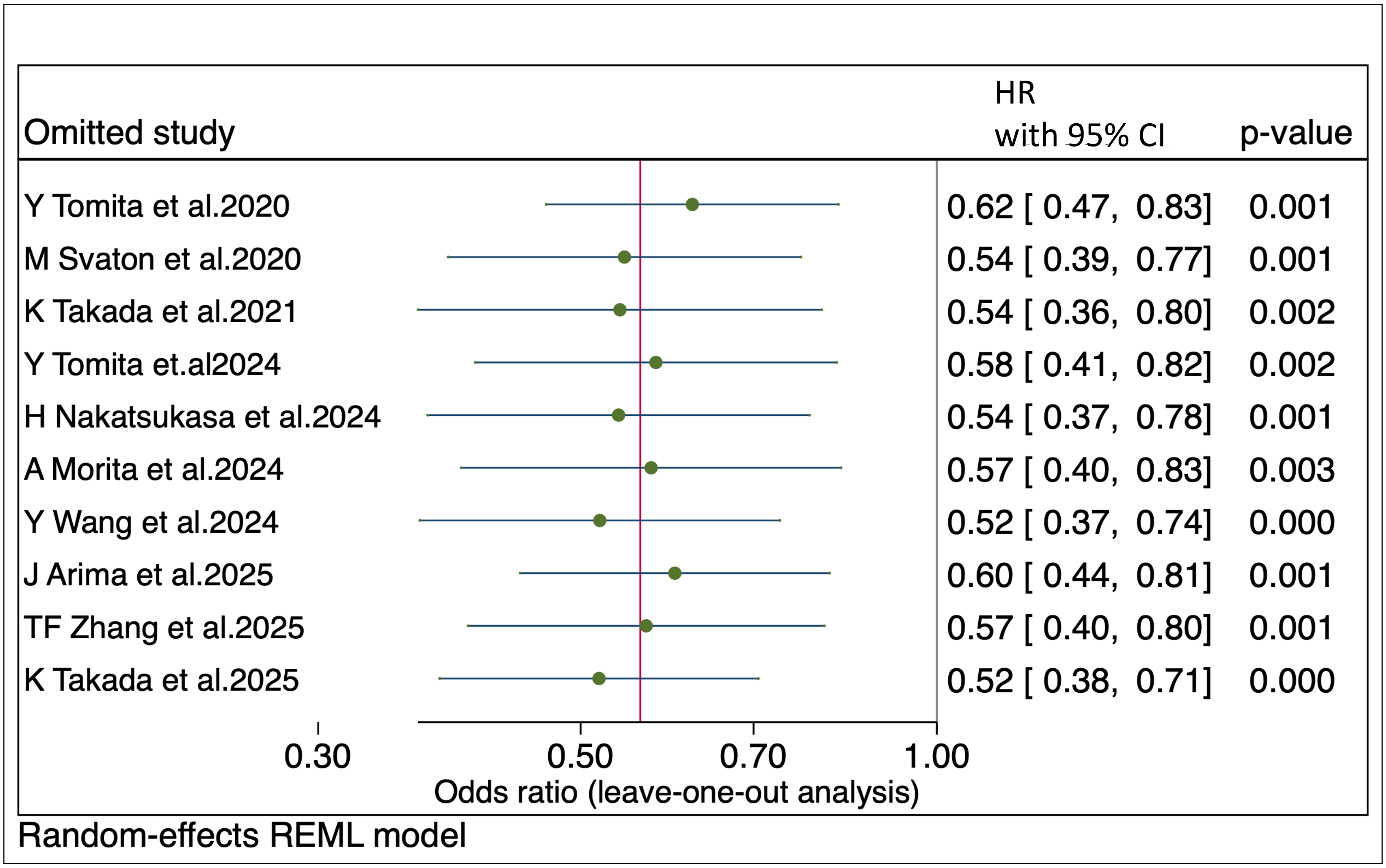

Figure S6. Leave-one-out sensitivity analysis of OS in patients receiving Probiotic using a random-effects model (REML). HR, hazard ratio; CI, confidence interval

Figure S7 Sensitivity analysis of PFS: Probiotics *vs.* No Probiotics

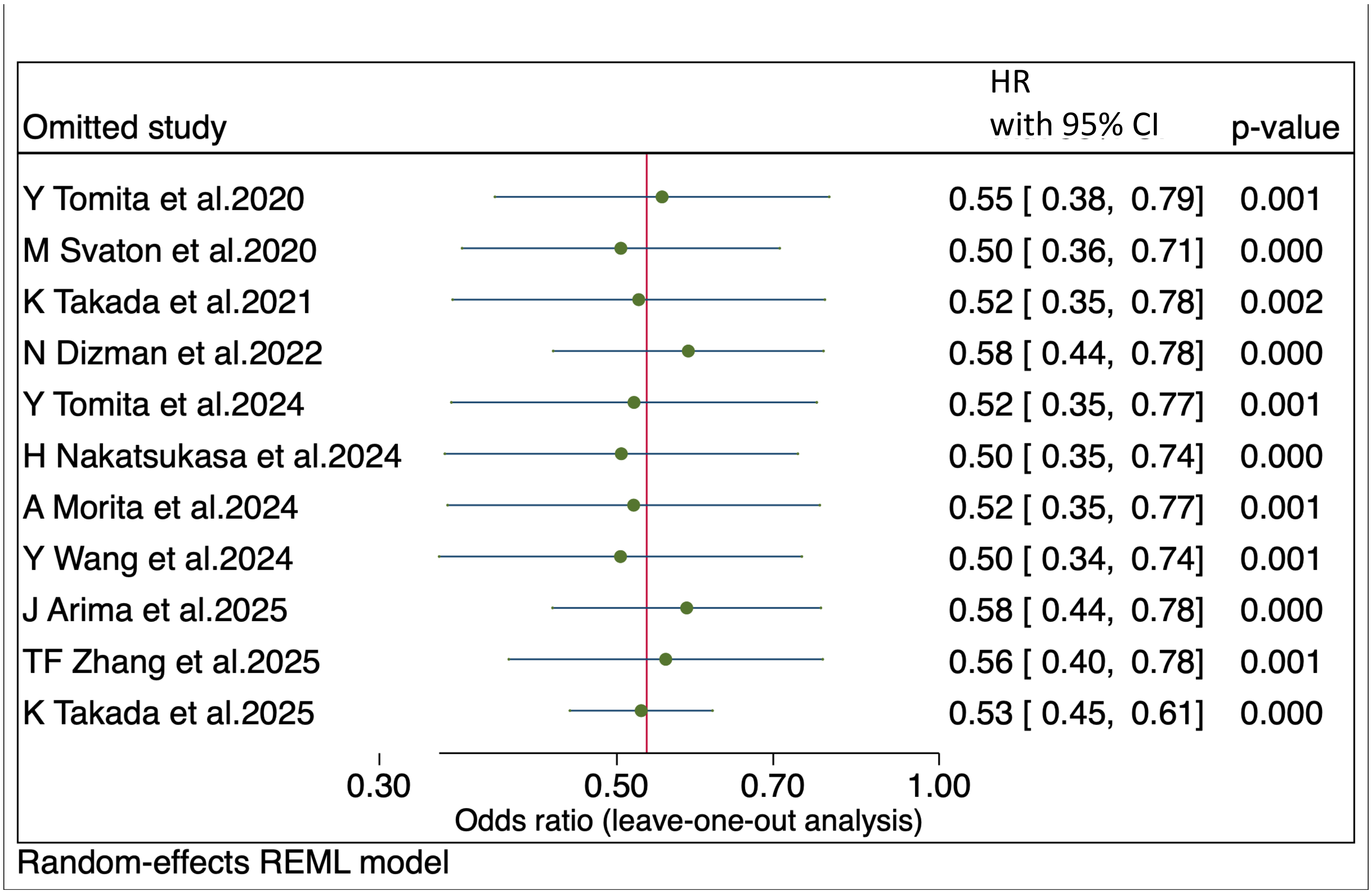

Figure S7. Leave-one-out sensitivity analysis of PFS in patients receiving Probiotic using a random-effects model (REML). HR, hazard ratio; CI, confidence interval

Figure S8 Funnel plot of ORR, OS and PFS: Probiotics *vs*. No Probiotics

A

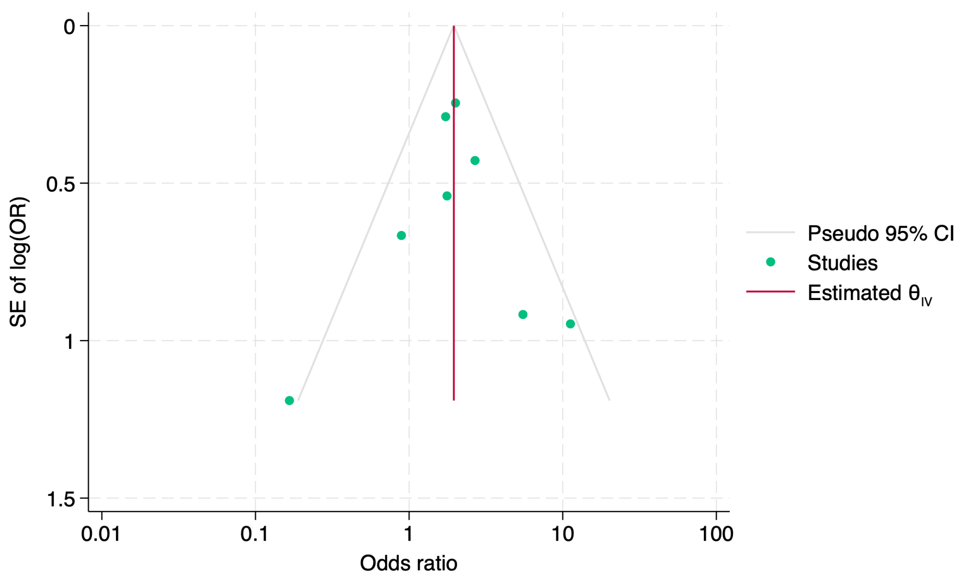

B

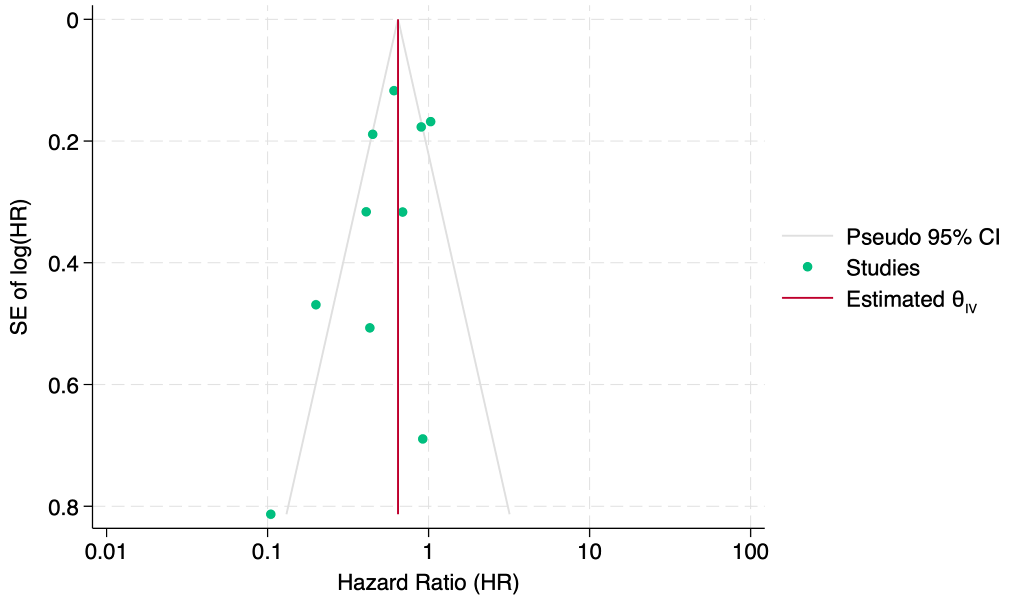

C

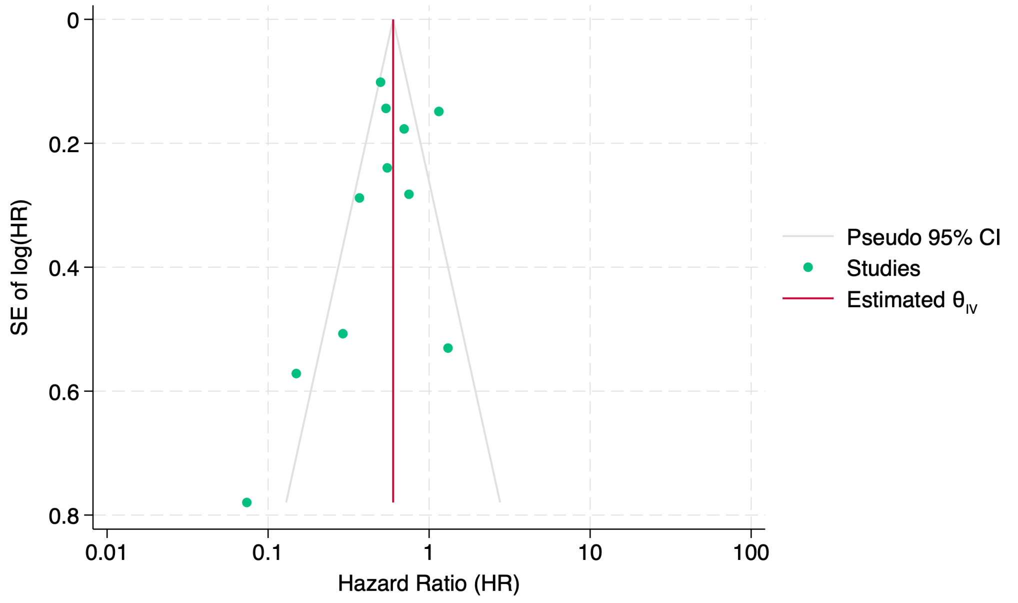

Figure S8. Funnel plot assessing publication bias for ORR **(A)**, OS **(B)** and PFS **(C)** in patients receiving Probiotics. ORR, objective response rate; OR, odds ratio; OS, overall survival; HR, hazard ratio, PFS, progression-free survival

Figure S9 Sensitivity analysis of ORR with FMT

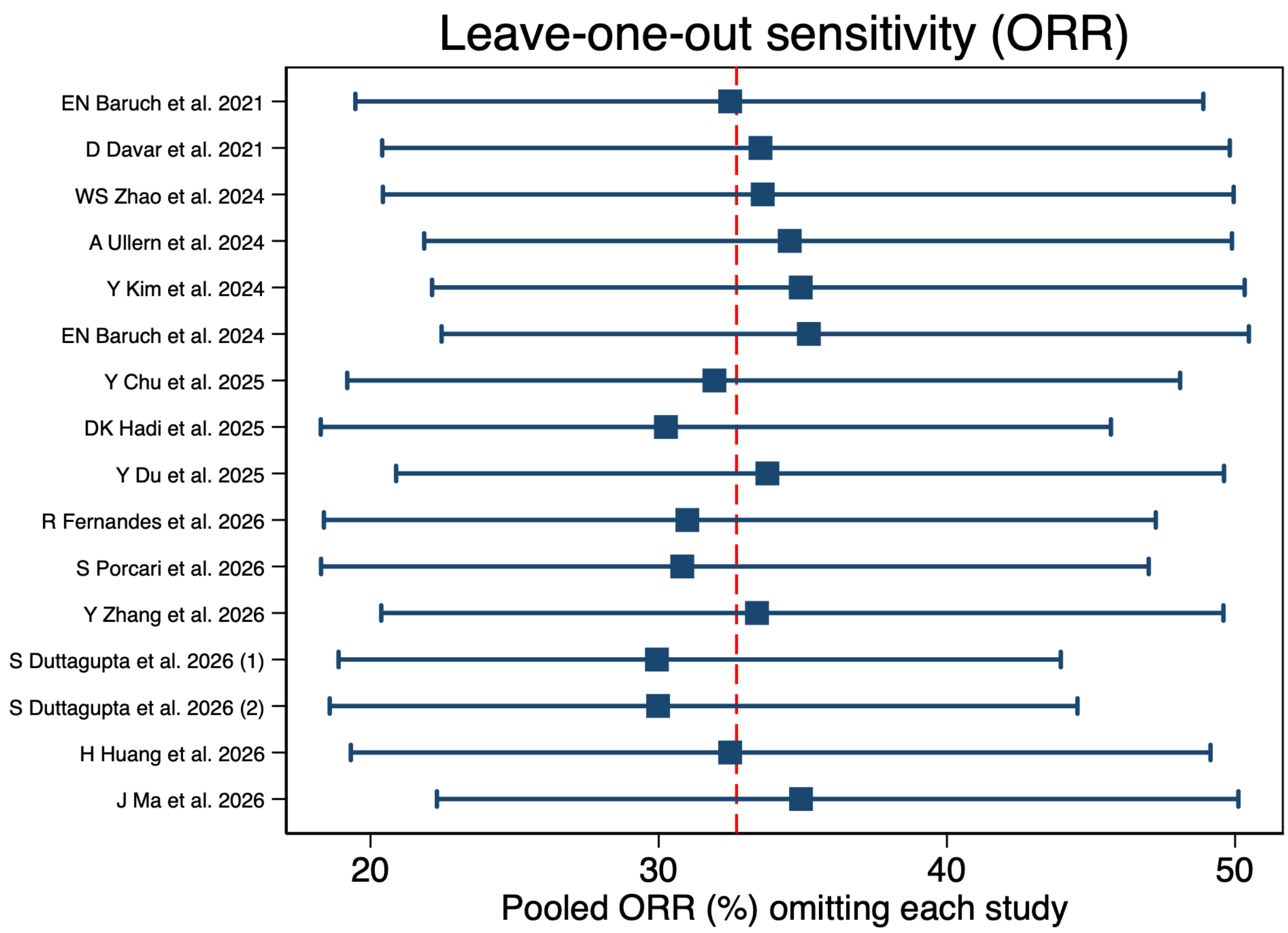

Figure S9. Leave-one-out sensitivity analysis of ORR in single-arm FMT studies using a random-effects model with logit transformation. ORR, objective response rate; CI, confidence interval; FMT, fecal microbiota transplantation.

Figure S10 Funnel plot of ORR with FMT

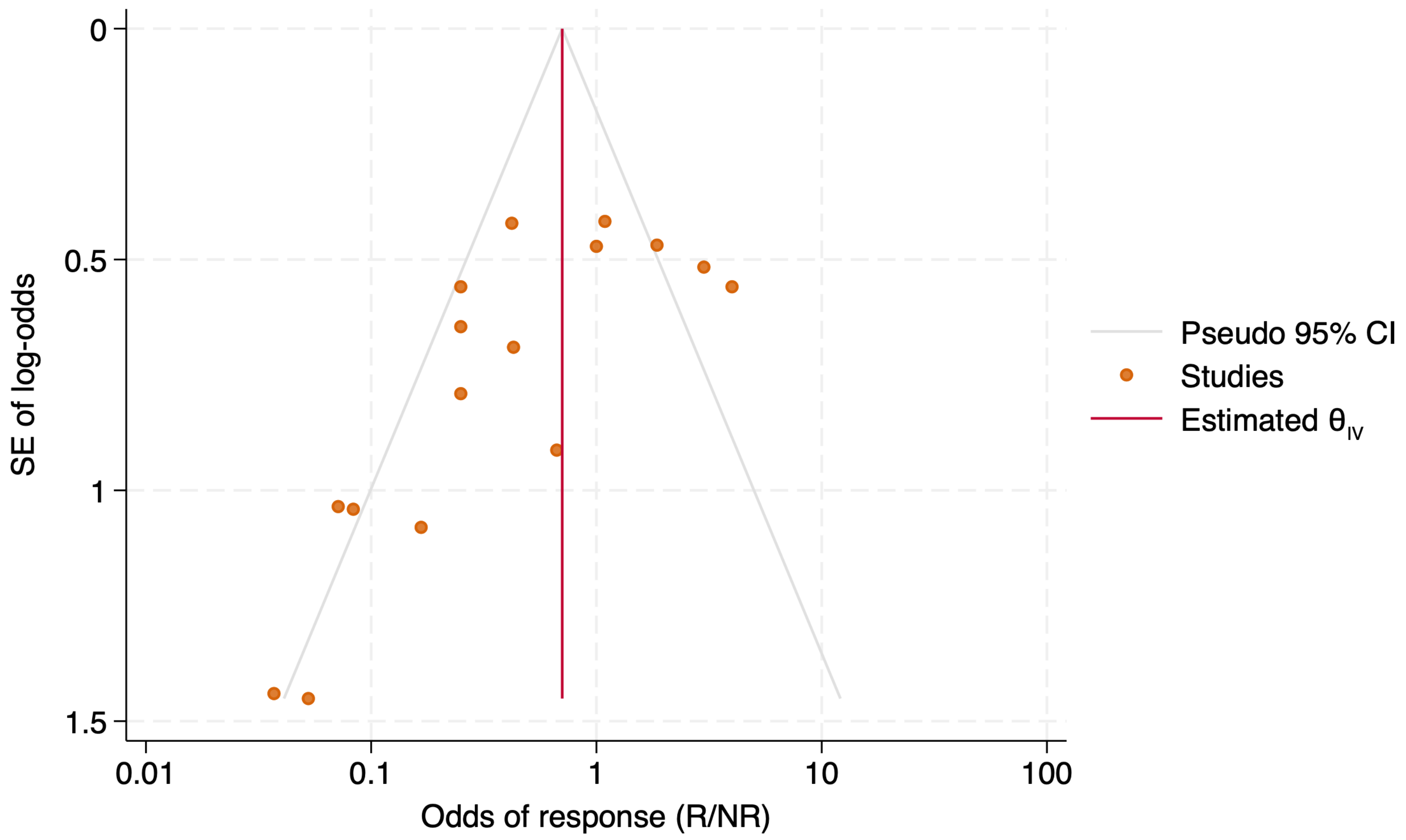

Figure S10. Funnel plot assessing publication bias for ORR in single-arm FMT studies based on logit-transformed ORR. ORR, objective response rate; FMT, fecal microbiota transplantation.

Figure S11 ROB of Antibiotic cohort graph and summary

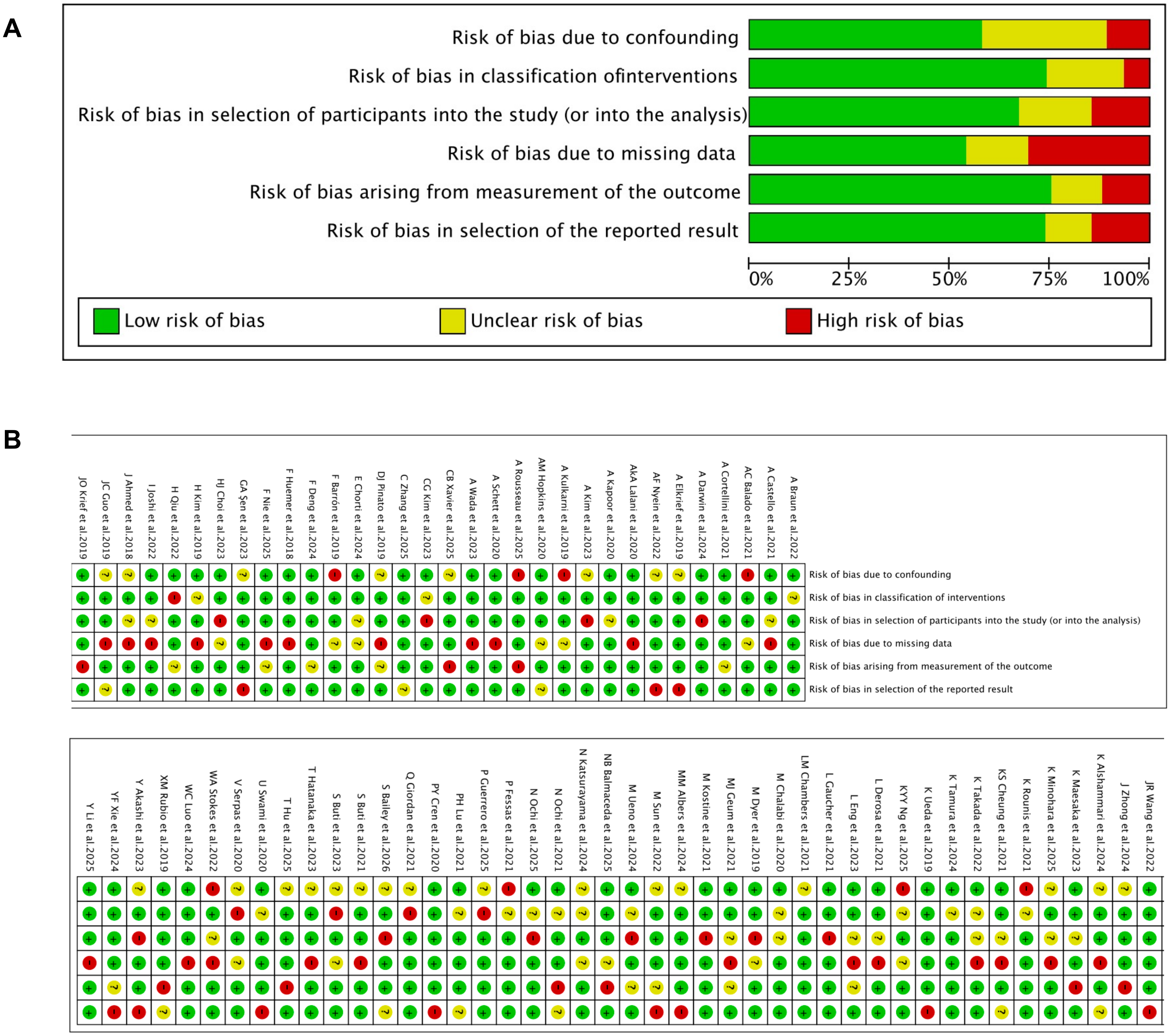

Figure S11. Risk of bias graph **(A)** and summary **(B)**: review authors' judgements about each risk of bias item presented as percentages across all included Antibiotic-related studies. ROB, risk of bias

Figure S12 ROB of FMT cohort graph and summary

A

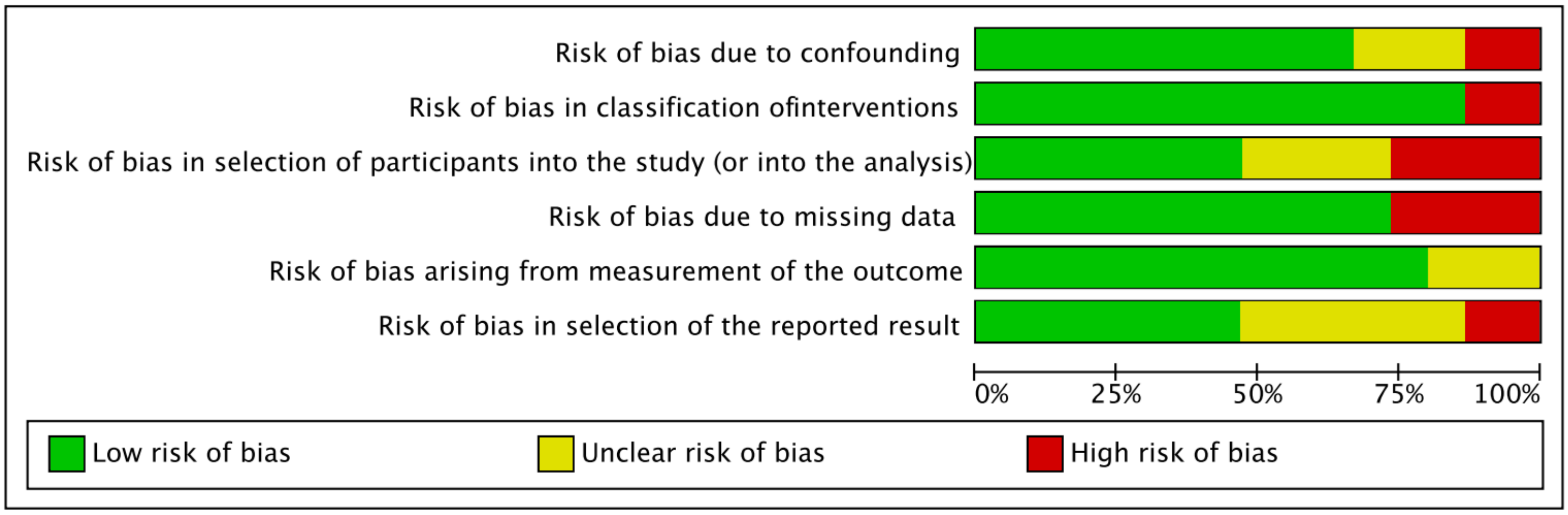

B

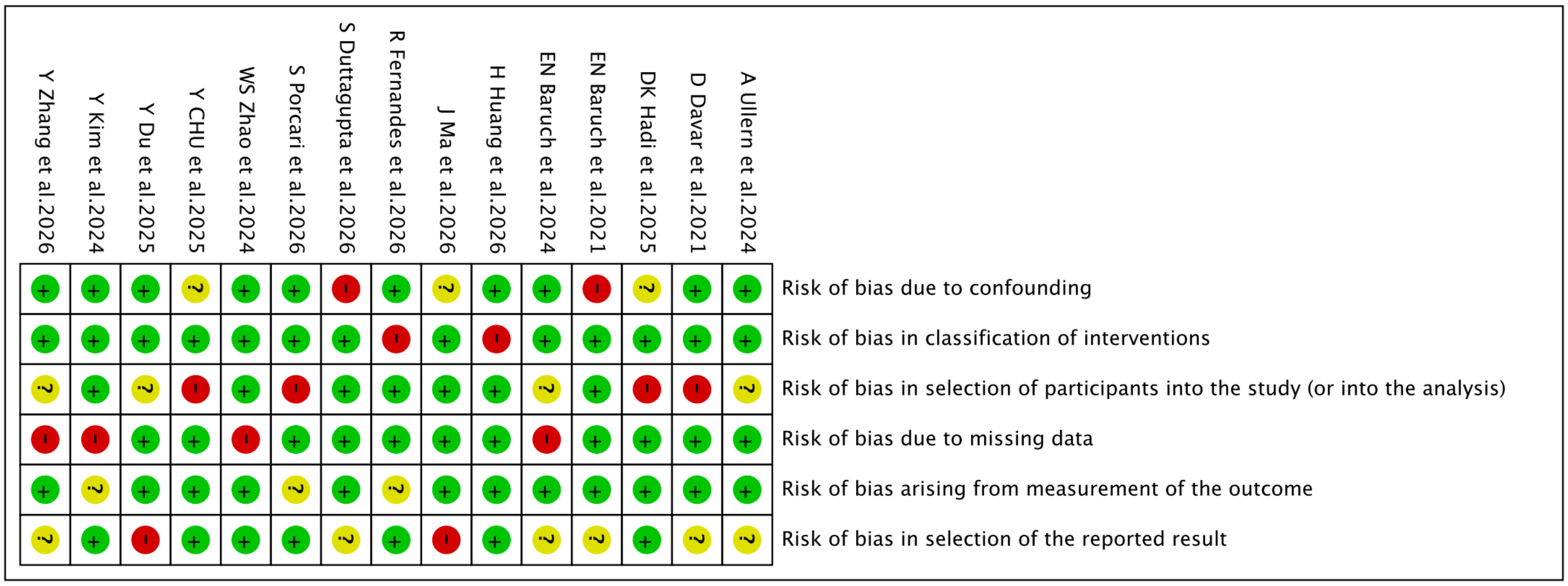

Figure S12. Risk of bias graph **(A)** and summary **(B)**: review authors' judgements about each risk of bias item presented as percentages across all included FMT-related studies. ROB, risk of bias; FMT, fecal microbiota transplantation

Figure S13 ROB of Probiotic cohort graph and summary

A

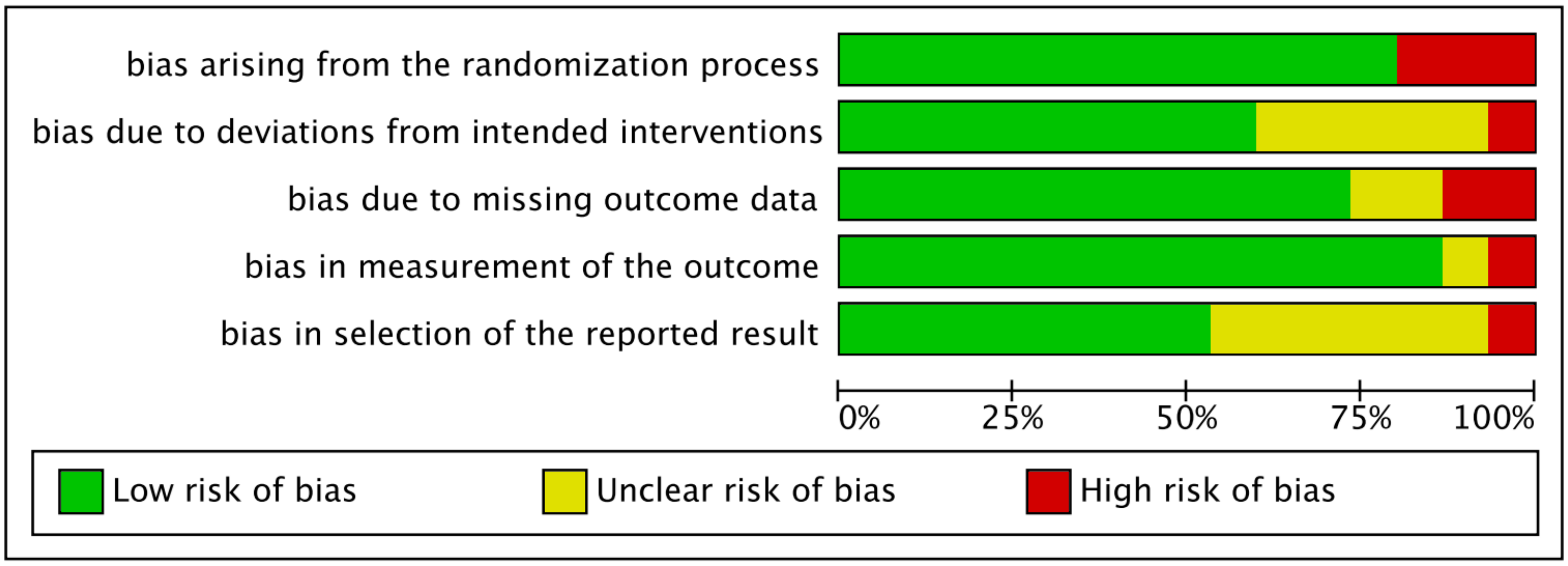

B

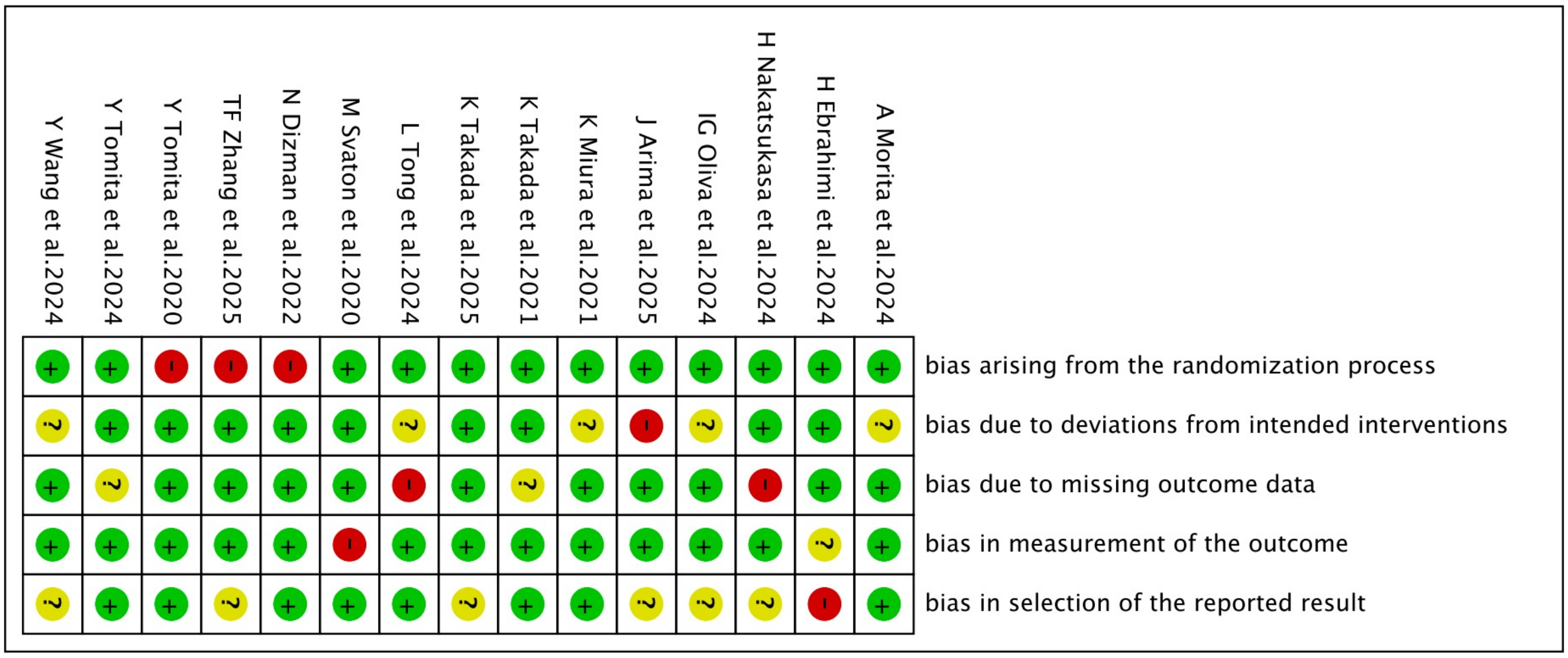

Figure S13. Risk of bias graph **(A)** and summary **(B)**: review authors' judgements about each risk of bias item presented as percentages across all included Probiotic-related studies. ROB, risk of bias
