# Supplementary material for: The use of antibiotic, probiotic, and fecal microbiota transplantation in modulating immunotherapy efficacy and survival: a systematic review and meta-analysis of clinical outcomes
Source: Oncologist. 2026 Jul 10;31(8):oyag264. doi: 10.1093/oncolo/oyag264 (PMC13401492; doi:10.1093/oncolo/oyag264)
Supplement: oyag264_Supplementary_Data [file oyag264_supplementary_data.zip › 20-Jul-2026_083856_Supplementary_Data.zip!/supplement_table.pdf]

Table S1. Search strategies used in PubMed

(neoplasms[Mesh] OR tumors[Title/Abstract] OR neoplasia)[Title/Abstract] OR neoplasias)[Title/Abstract] OR neoplasm)[Title/Abstract] OR tumor)[Title/Abstract] OR cancer)[Title/Abstract] OR cancers)[Title/Abstract] OR malignant neoplasm)[Title/Abstract] OR malignancy)[Title/Abstract] OR malignancies)[Title/Abstract] OR malignant neoplasms)[Title/Abstract] OR neoplasm, malignant)[Title/Abstract] OR neoplasms, malignant)[Title/Abstract] OR benign neoplasms)[Title/Abstract] OR neoplasms, benign)[Title/Abstract] OR neoplasm, benign)[Title/Abstract] OR benign neoplasm)[Title/Abstract] OR oncology)[Title/Abstract] OR carcinoma[Title/Abstract]) AND (immune checkpoint inhibitors[Mesh] OR immune checkpoint inhibitors[Title/Abstract] OR checkpoint inhibitors, immune[Title/Abstract] OR immune checkpoint blockers[Title/Abstract] OR checkpoint blockers, immune[Title/Abstract] OR immune checkpoint inhibitor[Title/Abstract] OR checkpoint inhibitor, immune[Title/Abstract] OR CTLA-4 inhibitors[Title/Abstract] OR CTLA 4 inhibitors[Title/Abstract] OR cytotoxic t-lymphocyte-associated protein 4 inhibitors[Title/Abstract] OR cytotoxic t-lymphocyte-associated protein 4 inhibitors[Title/Abstract] OR cytotoxic t-lymphocyte-associated protein 4 inhibitor[Title/Abstract] OR cytotoxic t lymphocyte associated protein 4 inhibitor[Title/Abstract] OR CTLA-4 inhibitor[Title/Abstract] OR CTLA 4 inhibitor[Title/Abstract] OR PD-1 inhibitors[Title/Abstract] OR PD 1 inhibitors[Title/Abstract] OR programmed cell death protein 1 inhibitor[Title/Abstract] OR programmed cell death protein 1 inhibitors[Title/Abstract] OR PD-1 inhibitor[Title/Abstract] OR inhibitor, PD-1[Title/Abstract] OR PD 1 inhibitor[Title/Abstract] OR immune checkpoint blockade[Title/Abstract] OR checkpoint blockade, immune[Title/Abstract] OR immune checkpoint inhibition[Title/Abstract] OR checkpoint inhibition, immune[Title/Abstract] OR PD-L1 inhibitors[Title/Abstract] OR PD L1 inhibitors[Title/Abstract] OR programmed death-ligand 1 inhibitors[Title/Abstract] OR programmed death ligand 1 inhibitors[Title/Abstract] OR PD-L1 inhibitor[Title/Abstract] OR PD L1 inhibitor[Title/Abstract] OR PD-1/PD-L1 blockade[Title/Abstract] OR blockade, PD-1/PD-L1[Title/Abstract] OR PD 1 PD L1 blockade[Title/Abstract] OR ICI[Title/Abstract] OR ICIs[Title/Abstract] OR nivolumab[Title/Abstract] OR pembrolizumab[Title/Abstract] OR avelumab[Title/Abstract] OR atezolizumab[Title/Abstract] OR tremelimumab[Title/Abstract] OR pidilizumab[Title/Abstract] OR durvalumab[Title/Abstract] OR lambrolizumab[Title/Abstract] OR cemiplimab[Title/Abstract] OR ipilimumab[Title/Abstract] OR camrelizumab[Title/Abstract] OR tislelizumab[Title/Abstract] OR sintilimab[Title/Abstract]) AND

("Anti-Bacterial Agents"[Mesh] OR Agents, Anti-Bacterial[Title/Abstract] OR Anti Bacterial Agents[Title/Abstract] OR Anti-Bacterial Compounds[Title/Abstract] OR Anti Bacterial Compounds[Title/Abstract] OR Compounds, Anti-Bacterial[Title/Abstract] OR Antibacterial Agents[Title/Abstract] OR Agents, Antibacterial[Title/Abstract] OR Anti-Bacterial Agent[Title/Abstract] OR Agent, Anti-Bacterial[Title/Abstract] OR Anti Bacterial Agent[Title/Abstract] OR Anti-Bacterial Compound[Title/Abstract] OR Anti Bacterial Compound[Title/Abstract] OR Compound, Anti-Bacterial[Title/Abstract] OR Antibacterial Agent[Title/Abstract] OR Agent, Antibacterial[Title/Abstract] OR Antibiotics[Title/Abstract] OR Antibiotic[Title/Abstract] OR Anti-Mycobacterial Agents[Title/Abstract] OR Agents, Anti-Mycobacterial[Title/Abstract] OR Anti Mycobacterial Agents[Title/Abstract] OR Antimycobacterial Agents[Title/Abstract] OR Agents, Antimycobacterial[Title/Abstract] OR Anti-Mycobacterial Agent[Title/Abstract] OR Agent, Anti-Mycobacterial[Title/Abstract] OR Anti Mycobacterial Agent[Title/Abstract] OR Antimycobacterial Agent[Title/Abstract] OR Agent, Antimycobacterial[Title/Abstract] OR Bacteriocidal Agents[Title/Abstract] OR Agents, Bacteriocidal[Title/Abstract] OR Bacteriocides[Title/Abstract] OR Bacteriocidal Agent[Title/Abstract] OR Agent, Bacteriocidal[Title/Abstract] OR Bacteriocide OR "Probiotics"[Mesh] OR Probiotic[Title/Abstract] OR "Prebiotics"[Mesh] OR Prebiotic[Title/Abstract] OR "Fecal Microbiota Transplantation"[Mesh] OR Fecal Microbiota Transplantations[Title/Abstract]OR Microbiota Transplantation, Fecal[Title/Abstract]OR Microbiota Transplantations, Fecal[Title/Abstract]OR Transplantation, Fecal Microbiota[Title/Abstract]OR Transplantations, Fecal Microbiota[Title/Abstract]OR Fecal Microbiota Transplant[Title/Abstract]OR Fecal Microbiota Transplants[Title/Abstract]OR Microbiota Transplant, Fecal[Title/Abstract]OR Microbiota Transplants, Fecal[Title/Abstract]OR Transplant, Fecal Microbiota[Title/Abstract]OR Transplants, Fecal Microbiota[Title/Abstract]OR Fecal Microbiome Transplantation[Title/Abstract]OR Fecal Microbiome Transplantations[Title/Abstract]OR Microbiome Transplantation, Fecal[Title/Abstract]OR Microbiome Transplantations, Fecal[Title/Abstract]OR Transplantation, Fecal Microbiome[Title/Abstract]OR Transplantations, Fecal Microbiome[Title/Abstract]OR Fecal Transplant[Title/Abstract]OR Fecal Transplants[Title/Abstract]OR Transplant, Fecal[Title/Abstract]OR Transplants, Fecal[Title/Abstract]OR Donor Feces Infusion[Title/Abstract]OR Donor Feces Infusions[Title/Abstract]OR Feces Infusion, Donor[Title/Abstract]OR Feces Infusions, Donor[Title/Abstract]OR Infusion, Donor Feces[Title/Abstract]OR Infusions, Donor

Feces[Title/Abstract]OR Fecal Transplantation[Title/Abstract]OR Fecal Transplantations[Title/Abstract]OR Transplantation, Fecal[Title/Abstract]OR Transplantations, Fecal[Title/Abstract]OR Intestinal Microbiota Transfer[Title/Abstract]OR Intestinal Microbiota Transfers[Title/Abstract]OR Microbiota Transfer, Intestinal[Title/Abstract]OR Microbiota Transfers, Intestinal[Title/Abstract]OR Transfer, Intestinal Microbiota[Title/Abstract]OR Transfers, Intestinal Microbiota[Title/Abstract]OR Intestinal Microbiota Transplantation[Title/Abstract]OR Intestinal Microbiota Transplantations[Title/Abstract]OR Microbiota Transplantation, Intestinal[Title/Abstract]OR Microbiota Transplantations, Intestinal[Title/Abstract]OR Transplantation, Intestinal Microbiota[Title/Abstract]OR Transplantations, Intestinal Microbiota[Title/Abstract]OR Intestinal Microbiome Transplantation[Title/Abstract]OR Intestinal Microbiome Transplantations[Title/Abstract]OR Microbiome Transplantation, Intestinal[Title/Abstract]OR Microbiome Transplantations, Intestinal[Title/Abstract]OR Transplantation, Intestinal Microbiome[Title/Abstract]OR Transplantations, Intestinal Microbiome[Title/Abstract]OR Intestinal Microbiota Transplant[Title/Abstract]OR Intestinal Microbiota Transplants[Title/Abstract]OR Microbiota Transplant, Intestinal[Title/Abstract]OR Microbiota Transplants, Intestinal[Title/Abstract]OR Transplant, Intestinal Microbiota[Title/Abstract]OR Transplants, Intestinal Microbiota[Title/Abstract]OR Intestinal Microbiome Transfer[Title/Abstract]OR Intestinal Microbiome Transfers[Title/Abstract]OR Microbiome Transfer, Intestinal[Title/Abstract]OR Microbiome Transfers, Intestinal[Title/Abstract]OR Transfer, Intestinal Microbiome[Title/Abstract]OR Transfers, Intestinal Microbiome[Title/Abstract]OR Fecal Microbiota Transfer[Title/Abstract]OR Fecal Microbiota Transfers[Title/Abstract]OR Microbiota Transfer, Fecal[Title/Abstract]OR Microbiota Transfers, Fecal[Title/Abstract]OR Transfer, Fecal Microbiota[Title/Abstract]OR Transfers, Fecal Microbiota[Title/Abstract]OR Intestinal Microbiome Transplant[Title/Abstract]OR Intestinal Microbiome Transplants[Title/Abstract]OR Microbiome Transplant, Intestinal[Title/Abstract]OR Microbiome Transplants, Intestinal[Title/Abstract]OR Transplant, Intestinal Microbiome[Title/Abstract]OR Transplants, Intestinal Microbiome[Title/Abstract])

Results：943

Table S2. Search strategies used in and Cochrane Library

	Search Terms/Phrases	Number
#1	MeSH descriptor: [Neoplasms] explode all trees	129459
#2	(tumors):ti,ab,kw OR (neoplasia):ti,ab,kw OR (neoplasias):ti,ab,kw OR (neoplasm):ti,ab,kw OR (tumor):ti,ab,kw OR (cancer):ti,ab,kw OR (cancers):ti,ab,kw OR (malignant neoplasm):ti,ab,kw OR (malignancy):ti,ab,kw OR (malignancies):ti,ab,kw OR (malignant neoplasms):ti,ab,kw OR (neoplasm, malignant):ti,ab,kw OR (neoplasms, malignant):ti,ab,kw OR (benign neoplasms):ti,ab,kw OR (neoplasms, benign):ti,ab,kw OR (neoplasm, benign):ti,ab,kw OR (benign neoplasm):ti,ab,kw OR (oncology):ti,ab,kw OR (carcinoma):ti,ab,kw	287683
#3	(#1) OR (#2)	307934
#4	MeSH descriptor: [Immune Checkpoint Inhibitors] explode all trees	400
#5	(immune checkpoint inhibitors):ti,ab,kw OR (checkpoint inhibitors, immune):ti,ab,kw OR (immune checkpoint blockers):ti,ab,kw OR (checkpoint blockers, immune):ti,ab,kw OR (immune checkpoint inhibitor):ti,ab,kw OR (checkpoint inhibitor, immune):ti,ab,kw OR (CTLA-4 inhibitors):ti,ab,kw OR (CTLA 4 inhibitors):ti,ab,kw OR (cytotoxic t-lymphocyte-associated protein 4 inhibitors):ti,ab,kw OR (cytotoxic t-lymphocyte-associated protein 4 inhibitors):ti,ab,kw OR (cytotoxic t-lymphocyte-associated protein 4 inhibitor):ti,ab,kw OR (cytotoxic t lymphocyte associated protein 4 inhibitor):ti,ab,kw OR (CTLA-4 inhibitor):ti,ab,kw OR (CTLA 4 inhibitor):ti,ab,kw OR (PD-1 inhibitors):ti,ab,kw OR (PD 1 inhibitors):ti,ab,kw OR (programmed cell death protein 1 inhibitor):ti,ab,kw OR (programmed cell death protein 1 inhibitors):ti,ab,kw OR (PD-1 inhibitor):ti,ab,kw OR (inhibitor, PD-1):ti,ab,kw OR (PD 1 inhibitor):ti,ab,kw OR (immune checkpoint blockade):ti,ab,kw OR (checkpoint blockade, immune):ti,ab,kw OR (immune checkpoint inhibition):ti,ab,kw OR (checkpoint inhibition, immune):ti,ab,kw OR (PD-L1 inhibitors):ti,ab,kw OR (PD L1 inhibitors):ti,ab,kw OR (programmed death-ligand 1 inhibitors):ti,ab,kw OR (programmed death ligand 1 inhibitors):ti,ab,kw OR (PD-L1 inhibitor):ti,ab,kw OR (PD L1 inhibitor):ti,ab,kw OR (PD-1/PD-L1	18796

	blockade):ti,ab,kw OR (blockade, PD-1/PD-L1):ti,ab,kw OR (PD 1 PD L1 blockade):ti,ab,kw OR (ICI):ti,ab,kw OR (ICIs):ti,ab,kw OR (nivolumab):ti,ab,kw OR (pembrolizumab):ti,ab,kw OR (avelumab):ti,ab,kw OR (atezolizumab):ti,ab,kw OR (tremelimumab):ti,ab,kw OR (pidilizumab):ti,ab,kw OR (durvalumab):ti,ab,kw OR (lambrolizumab):ti,ab,kw OR (cemiplimab):ti,ab,kw OR (ipilimumab):ti,ab,kw OR (camrelizumab):ti,ab,kw OR (tislelizumab):ti,ab,kw OR (sintilimab):ti,ab,kw	
#6	(#4) OR (#5)	18796
#7	MeSH descriptor: [Anti-Bacterial Agents] explode all trees	17002
#8	(Agents, Anti-Bacterial):ti,ab,kw OR (Anti Bacterial Agents):ti,ab,kw OR (Anti-Bacterial Compounds):ti,ab,kw OR (Anti Bacterial Compounds):ti,ab,kw OR (Compounds, Anti-Bacterial):ti,ab,kw OR (Antibacterial Agents):ti,ab,kw OR (Agents, Antibacterial):ti,ab,kw OR (Anti-Bacterial Agent):ti,ab,kw OR (Agent, Anti-Bacterial):ti,ab,kw OR (Anti Bacterial Agent):ti,ab,kw OR (Anti-Bacterial Compound):ti,ab,kw OR (Anti Bacterial Compound):ti,ab,kw OR (Compound, Anti-Bacterial):ti,ab,kw OR (Antibacterial Agent):ti,ab,kw OR (Agent, Antibacterial):ti,ab,kw OR (Antibiotics):ti,ab,kw OR (Antibiotic):ti,ab,kw OR (Anti-Mycobacterial Agents):ti,ab,kw OR (Agents, Anti-Mycobacterial):ti,ab,kw OR (Anti Mycobacterial Agents):ti,ab,kw OR (Antimycobacterial Agents):ti,ab,kw OR (Agents, Antimycobacterial):ti,ab,kw OR (Anti-Mycobacterial Agent):ti,ab,kw OR (Agent, Anti-Mycobacterial):ti,ab,kw OR (Anti Mycobacterial Agent):ti,ab,kw OR (Antimycobacterial Agent):ti,ab,kw OR (Agent, Antimycobacterial):ti,ab,kw OR (Bacteriocidal Agents):ti,ab,kw OR (Agents, Bacteriocidal):ti,ab,kw OR (Bacteriocides):ti,ab,kw OR (Bacteriocidal Agent):ti,ab,kw OR (Agent, Bacteriocidal):ti,ab,kw OR (Bacteriocide):ti,ab,kw	48975
#9	(#7) OR (#8)	50268
#10	MeSH descriptor: [Probiotics] explode all trees	3702
#11	(Probiotic):ti,ab,kw	8207

#12	(#10) OR (#11)	9263
#13	MeSH descriptor: [Prebiotics] explode all trees	758
#14	(Prebiotic):ti,ab,kw	1705
#15	(#13) OR (#14)	2064
#16	MeSH descriptor: [Fecal Microbiota Transplantation] explode all trees	264
#17	(Fecal Microbiota Transplantations):ti,ab,kw OR (Microbiota Transplantation, Fecal):ti,ab,kw OR (Microbiota Transplantations, Fecal):ti,ab,kw OR (Transplantation, Fecal Microbiota):ti,ab,kw OR (Transplantations, Fecal Microbiota):ti,ab,kw OR (Fecal Microbiota Transplant):ti,ab,kw OR (Fecal Microbiota Transplants):ti,ab,kw OR (Microbiota Transplant, Fecal):ti,ab,kw OR (Microbiota Transplants, Fecal):ti,ab,kw OR (Transplant, Fecal Microbiota):ti,ab,kw OR (Transplants, Fecal Microbiota):ti,ab,kw OR (Fecal Microbiome Transplantation):ti,ab,kw OR (Fecal Microbiome Transplantations):ti,ab,kw OR (Microbiome Transplantation, Fecal):ti,ab,kw OR (Microbiome Transplantations, Fecal):ti,ab,kw OR (Transplantation, Fecal Microbiome):ti,ab,kw OR (Transplantations, Fecal Microbiome):ti,ab,kw OR (Fecal Transplant):ti,ab,kw OR (Fecal Transplants):ti,ab,kw OR (Transplant, Fecal):ti,ab,kw OR (Transplants, Fecal):ti,ab,kw OR (Fecal Transplantation):ti,ab,kw OR (Fecal Transplantations):ti,ab,kw OR (Transplantation, Fecal):ti,ab,kw OR (Transplantations, Fecal):ti,ab,kw OR (Intestinal Microbiota Transfer):ti,ab,kw OR (Intestinal Microbiota Transfers):ti,ab,kw OR (Microbiota Transfer, Intestinal):ti,ab,kw OR (Microbiota Transfers, Intestinal):ti,ab,kw OR (Transfer, Intestinal Microbiota):ti,ab,kw OR (Transfers, Intestinal Microbiota):ti,ab,kw OR (Intestinal Microbiota Transplantation):ti,ab,kw OR (Intestinal Microbiota Transplantations):ti,ab,kw OR (Microbiota Transplantation, Intestinal):ti,ab,kw OR (Microbiota Transplantations, Intestinal):ti,ab,kw OR (Transplantation, Intestinal Microbiota):ti,ab,kw OR (Transplantations, Intestinal Microbiota):ti,ab,kw OR (Intestinal Microbiome Transplantation):ti,ab,kw OR (Intestinal Microbiome Transplantations):ti,ab,kw	1467

	OR (Microbiome Transplantation, Intestinal):ti,ab,kw OR (Microbiome Transplantations, Intestinal):ti,ab,kw OR (Transplantation, Intestinal Microbiome):ti,ab,kw OR (Transplantations, Intestinal Microbiome):ti,ab,kw OR (Intestinal Microbiota Transplant):ti,ab,kw OR (Intestinal Microbiota Transplants):ti,ab,kw OR (Microbiota Transplant, Intestinal):ti,ab,kw OR (Microbiota Transplants, Intestinal):ti,ab,kw OR (Transplant, Intestinal Microbiota):ti,ab,kw OR (Transplants, Intestinal Microbiota):ti,ab,kw OR (Intestinal Microbiome Transfer):ti,ab,kw OR (Intestinal Microbiome Transfers):ti,ab,kw OR (Microbiome Transfer, Intestinal):ti,ab,kw OR (Microbiome Transfers, Intestinal):ti,ab,kw OR (Transfer, Intestinal Microbiome):ti,ab,kw OR (Transfers, Intestinal Microbiome):ti,ab,kw OR (Fecal Microbiota Transfer):ti,ab,kw OR (Fecal Microbiota Transfers):ti,ab,kw OR (Microbiota Transfer, Fecal):ti,ab,kw OR (Microbiota Transfers, Fecal):ti,ab,kw OR (Transfer, Fecal Microbiota):ti,ab,kw OR (Transfers, Fecal Microbiota):ti,ab,kw OR (Intestinal Microbiome Transplant):ti,ab,kw OR (Intestinal Microbiome Transplants):ti,ab,kw OR (Microbiome Transplant, Intestinal):ti,ab,kw OR (Microbiome Transplants, Intestinal):ti,ab,kw OR (Transplant, Intestinal Microbiome):ti,ab,kw OR (Transplants, Intestinal Microbiome):ti,ab,kw	
#18	(#16) OR (#17)	1467
#19	(#9) OR (#12) OR (#15) OR (#18)	60230
#20	(#3) AND (#6) AND (#19)	108

Table S3. Search strategies used in EMBASE

	Search Terms/Phrases	Number
#1	'neoplasm'/exp OR 'neoplasm'	6,891,074
#2	'tumors':ab,kw,ti OR 'neoplasia':ab,kw,ti OR 'neoplasias':ab,kw,ti OR 'neoplasm':ab,kw,ti OR 'tumor':ab,kw,ti OR 'cancer':ab,kw,ti OR 'cancers':ab,kw,ti OR 'malignant neoplasm':ab,kw,ti OR 'malignancy':ab,kw,ti OR 'malignancies':ab,kw,ti OR 'malignant neoplasms':ab,kw,ti OR 'neoplasm, malignant':ab,kw,ti OR 'neoplasms, malignant':ab,kw,ti OR 'benign neoplasms':ab,kw,ti OR 'neoplasms, benign':ab,kw,ti OR 'neoplasm, benign':ab,kw,ti OR 'benign neoplasm':ab,kw,ti OR 'oncology':ab,kw,ti OR 'carcinoma':ab,kw,ti	5,935807
#3	#1 OR #2	7985541
#4	'immune checkpoint inhibitor'/exp	48391
#5	'immune checkpoint inhibitors':ab,kw,ti OR 'checkpoint inhibitors, immune':ab,kw,ti OR 'immune checkpoint blockers':ab,kw,ti OR 'checkpoint blockers, immune':ab,kw,ti OR 'immune checkpoint inhibitor':ab,kw,ti OR 'checkpoint inhibitor, immune':ab,kw,ti OR 'ctla-4 inhibitors':ab,kw,ti OR 'ctla 4 inhibitors':ab,kw,ti OR 'cytotoxic t-lymphocyte-associated protein 4 inhibitors':ab,kw,ti OR 'cytotoxic t-lymphocyte-associated protein 4 inhibitor':ab,kw,ti OR 'cytotoxic t lymphocyte associated protein 4 inhibitor':ab,kw,ti OR 'ctla-4 inhibitor':ab,kw,ti OR 'ctla 4 inhibitor':ab,kw,ti OR 'pd-1 inhibitors':ab,kw,ti OR 'pd 1 inhibitors':ab,kw,ti OR 'programmed cell death protein 1 inhibitor':ab,kw,ti OR 'programmed cell death protein 1 inhibitors':ab,kw,ti OR 'pd-1 inhibitor':ab,kw,ti OR 'inhibitor, pd-1':ab,kw,ti OR 'pd 1 inhibitor':ab,kw,ti OR 'immune checkpoint blockade':ab,kw,ti OR 'checkpoint blockade, immune':ab,kw,ti OR 'immune checkpoint inhibition':ab,kw,ti OR 'checkpoint inhibition, immune':ab,kw,ti OR 'pd-l1 inhibitors':ab,kw,ti OR 'pd l1 inhibitors':ab,kw,ti OR 'programmed death-ligand 1 inhibitors':ab,kw,ti OR 'programmed death ligand 1 inhibitors':ab,kw,ti OR 'pd-l1 inhibitor':ab,kw,ti OR 'pd l1 inhibitor':ab,kw,ti OR 'pd-1/pd-l1 blockade':ab,kw,ti OR 'blockade, pd-1/pd-l1':ab,kw,ti OR 'pd 1 pd l1 blockade':ab,kw,ti OR 'ici':ab,kw,ti OR 'icis':ab,kw,ti OR 'nivolumab':ab,kw,ti OR 'pembrolizumab':ab,kw,ti OR 'avelumab':ab,kw,ti OR 'atezolizumab':ab,kw,ti OR 'tremelimumab':ab,kw,ti OR 'pidilizumab':ab,kw,ti OR 'durvalumab':ab,kw,ti OR 'lambrolizumab':ab,kw,ti OR 'cemiplimab':ab,kw,ti OR 'ipilimumab':ab,kw,ti OR 'camrelizumab':ab,kw,ti OR 'tislelizumab':ab,kw,ti OR 'sintilimab':ab,kw,ti	143356
#6	#4 OR #5	154664
#7	'agents, anti-bacterial':ab,kw,ti OR 'anti bacterial agents':ab,kw,ti OR 'anti-bacterial compounds':ab,kw,ti OR 'anti bacterial compounds':ab,kw,ti OR 'compounds, anti-bacterial':ab,kw,ti OR 'antibacterial agents':ab,kw,ti OR 'agents, antibacterial':ab,kw,ti OR 'anti-bacterial agent':ab,kw,ti OR 'agent, anti-bacterial':ab,kw,ti OR 'anti bacterial agent':ab,kw,ti OR 'anti-bacterial compound':ab,kw,ti OR 'anti bacterial compound':ab,kw,ti OR 'compound, anti-bacterial':ab,kw,ti OR 'antibacterial agent':ab,kw,ti OR 'agent,	685926

	antibacterial':ab,kw,ti OR 'antibiotics':ab,kw,ti OR 'antibiotic':ab,kw,ti OR 'anti-mycobacterial agents':ab,kw,ti OR 'agents, anti-mycobacterial':ab,kw,ti OR 'anti mycobacterial agents':ab,kw,ti OR 'antimycobacterial agents':ab,kw,ti OR 'agents, antimycobacterial':ab,kw,ti OR 'anti-mycobacterial agent':ab,kw,ti OR 'agent, anti-mycobacterial':ab,kw,ti OR 'anti mycobacterial agent':ab,kw,ti OR 'antimycobacterial agent':ab,kw,ti OR 'agent, antimycobacterial':ab,kw,ti OR 'bacteriocidal agents':ab,kw,ti OR 'agents, bacteriocidal':ab,kw,ti OR 'bacteriocides':ab,kw,ti OR 'bacteriocidal agent':ab,kw,ti OR 'agent, bacteriocidal':ab,kw,ti OR 'bacteriocide':ab,kw,ti	
#8	'probiotic agent'/exp	69918
#9	'probiotic':ab,kw,ti	41863
#10	#8 OR #9	77334
#11	'prebiotic agent'/exp	17461
#12	'prebiotic':ti,ab,kw	13364
#13	#11 OR #12	23047
#14	'fecal microbiota transplantation'/exp OR 'fecal microbiota transplantation'	15669
#15	'fecal microbiota transplantations':ab,kw,ti OR 'microbiota transplantation, fecal':ab,kw,ti OR 'microbiota transplantations, fecal':ab,kw,ti OR 'transplantation, fecal microbiota':ab,kw,ti OR 'transplantations, fecal microbiota':ab,kw,ti OR 'fecal microbiota transplant':ab,kw,ti OR 'fecal microbiota transplants':ab,kw,ti OR 'microbiota transplant, fecal':ab,kw,ti OR 'microbiota transplants, fecal':ab,kw,ti OR 'transplant, fecal microbiota':ab,kw,ti OR 'transplants, fecal microbiota':ab,kw,ti OR 'fecal microbiome transplantation':ab,kw,ti OR 'fecal microbiome transplantations':ab,kw,ti OR 'microbiome transplantation, fecal':ab,kw,ti OR 'microbiome transplantations, fecal':ab,kw,ti OR 'transplantation, fecal microbiome':ab,kw,ti OR 'transplantations, fecal microbiome':ab,kw,ti OR 'fecal transplant':ab,kw,ti OR 'fecal transplants':ab,kw,ti OR 'transplant, fecal':ab,kw,ti OR 'transplants, fecal':ab,kw,ti OR 'fecal transplantation':ab,kw,ti OR 'fecal transplantations':ab,kw,ti OR 'transplantation, fecal':ab,kw,ti OR 'transplantations, fecal':ab,kw,ti OR 'intestinal microbiota transfer':ab,kw,ti OR 'intestinal microbiota transfers':ab,kw,ti OR 'microbiota transfer, intestinal':ab,kw,ti OR 'microbiota transfers, intestinal':ab,kw,ti OR 'transfer, intestinal microbiota':ab,kw,ti OR 'transfers, intestinal microbiota':ab,kw,ti OR 'intestinal microbiota transplantation':ab,kw,ti OR 'intestinal microbiota transplantations':ab,kw,ti OR 'microbiota transplantation, intestinal':ab,kw,ti OR 'microbiota transplantations, intestinal':ab,kw,ti OR 'transplantation, intestinal microbiota':ab,kw,ti OR 'transplantations, intestinal microbiota':ab,kw,ti OR 'intestinal microbiome transplantation':ab,kw,ti OR 'intestinal microbiome transplantations':ab,kw,ti OR 'microbiome transplantation, intestinal':ab,kw,ti OR 'microbiome transplantations, intestinal':ab,kw,ti OR 'transplantation, intestinal microbiome':ab,kw,ti OR 'transplantations, intestinal microbiome':ab,kw,ti OR 'intestinal microbiota transplant':ab,kw,ti OR 'intestinal microbiota transplants':ab,kw,ti OR 'microbiota transplant, intestinal':ab,kw,ti OR 'microbiota transplants, intestinal':ab,kw,ti OR 'transplant, intestinal	3331

	microbiota':ab,kw,ti OR 'transplants, intestinal microbiota':ab,kw,ti OR 'intestinal microbiome transfer':ab,kw,ti OR 'intestinal microbiome transfers':ab,kw,ti OR 'microbiome transfer, intestinal':ab,kw,ti OR 'microbiome transfers, intestinal':ab,kw,ti OR 'transfer, intestinal microbiome':ab,kw,ti OR 'transfers, intestinal microbiome':ab,kw,ti OR 'fecal microbiota transfer':ab,kw,ti OR 'fecal microbiota transfers':ab,kw,ti OR 'microbiota transfer, fecal':ab,kw,ti OR 'microbiota transfers, fecal':ab,kw,ti OR 'transfer, fecal microbiota':ab,kw,ti OR 'transfers, fecal microbiota':ab,kw,ti OR 'intestinal microbiome transplant':ab,kw,ti OR 'intestinal microbiome transplants':ab,kw,ti OR 'microbiome transplant, intestinal':ab,kw,ti OR 'microbiome transplants, intestinal':ab,kw,ti OR 'transplant, intestinal microbiome':ab,kw,ti OR 'transplants, intestinal microbiome':ab,kw,ti	
#16	#14 OR #15	16293
#17	#7 OR #10 OR #13 OR #16	771718
#18	#3 AND #6 AND #17	2060

Table S4. Detail characteristics of 76 antibiotic cohort included in the meta-analysis

Author	Year	Country	Study name	Trial type	Median follow-up	Cancer Type	N	Line of Therapy	Antibiotics regimen in relation to ICI	Time frame	Immunotherapy	Antibiotic
F Huemer et al.	2018	Austria	-	Retrospective single-center study	-	NSCLC	30	≥ 2nd-line	1 month before ICI to 1 month after ICI	2015.5-2017.11	PD-1	Penicillins, fluoroquinolones, carbapenems
J Ahmed et al.	2018	USA	-	Retrospective cohort study	-	Various advanced solid tumors	60	1st-line or 2nd-line	ATB used within 2 weeks before or after ICI start; additional analysis for ATB use within 10 weeks before progression.	2014.4-2017.12	PD-1+PD-L1	Narrow-spectrum+broad-spectrum
A Elkrief et al.	2019	Canada	NCT01676649	Retrospective cohort study; some patients were treated as part of NCT01676649	-	Advanced/Stage IV melanoma	74	Mixed lines	ATB limited to 30 days before ICI start	- 2018.5.1	PD-1+CTLA-4	Mainly β-lactams ± β-lactamase inhibitors
A Kulkarni et al.	2019	USA	-	Retrospective single-center rstudy	-	NSCLC, RCC	148	-	Within 1 month before ICI or during ICI therapy	2015.5-2019.1	PD-1+PD-L1	-
DJ Pinato et al.	2019	UK	-	Prospective observational cohort study	-	Mixed solid tumors (NSCLC 119, melanoma 38, others 39)	196	-	Within 30 days before ICI and during ICI therapy	2015.1-2018.4	PD-1+PD-L1±CTLA-4	Mainly broad-spectrum β-lactams

F Barrón et al.	2019	Multiple Latin American countries	CLICAP- ABs	Retrospective study	-	Advanced NSCLC	140	-	Within 30 days prior to ICI initiation	-	PD-L1	Mostly β-lactams, quinolones
H Kim et al.	2019	Korea	-	Retrospective study	-	Multiple solid tumors (NSCLC most common: 56%)	234	Mixed lines	Antibiotic use within 60 days prior to ICI initiation	2012.2-2018.5	PD-1+PD-L1+CTLA-4	Cephalosporins 35%, fluoroquinolones 24%, β-lactams 17%, others 24%
JO Krief et al.	2019	France	-	Retrospective cohort study	500 days (16.4months)	Advanced NSCLC	72	Mostly≥ 2nd-line	From 2 months before to 1 month after nivolumab initiation	2014.7-2017.9	PD-1	Mostly β-lactams
JC Guo et al.	2019	Taiwan, China	-	Retrospective dual-center study	6.4 months（2.2-29.9）	ESCC	49	Mixed lines	Antibiotic exposure within 2 months before to 1 month after initiating ICI therapy	2015.8-2017.12	PD-1+PD-L1	Systemic antibiotics; mainly β-lactam
K Ueda et al.	2019	Japan	-	Retrospective cohort study	-	mRCC	31	Mixed lines	Antibiotic use within 30 days before first ICI dose	2016.11-2019.4	PD-1+CTLA-4	β-lactam antibiotics
M Dyer et al.	2019	Australia	Melanoma Research Victori	Retrospective study	-	Advanced melanoma	121	-	Within 30 days of the first ICI dose	2012.8-2018.5	-	-

			a Project									
XM Rubio et al.	2019	Spain	-	Retrospective multicenter study	6.5 months	Advanced NSCLC, PD-L1 ≥50%	121	1st-line	Antibiotics 2 months before to 1 month after ICI start	2016.9-2019.3	PD-1	Quinolones (40.7%), penicillins/derivatives (35.2%)
A Kapoor et al.	2020	India	-	Retrospective single-center real-world audit of a prospectively collected database	9.5 months	Multiple solid tumors, mainly NSCLC (63%) and HNC (15.8%), urinary tract (10%), RCC (8.6%)	155	Mixed lines	2 weeks before ICI to during ICI	2015.8-2018.11	PD-1	β-lactams, aminoglycosides, fluoroquinolones, carbapenems
A Schett et al.	2020	Switzerland (two tertiary centers)	-	Retrospective observational cohort study	-	Advanced NSCLC (EGFR WT, ALK-)	218	Mixed lines	Within 2 months prior to ICI start	2013 .1-2017. 12	PD-1+PD-L1	β-lactams, quinolones, macrolides
AKA Lalani et al.	2020	USA	-	Retrospective cohort study	16.6 months	mRCC	146	Mixed therapy lines: 1st-line 43.2%, 2nd-line 26.7%,	8 weeks before to 4 weeks after initiation	2009-2017	PD-1+PD-L1+CTLA-4	β-lactam, fluoroquinolones, macrolides, tetracyclines

								≥3rd-line 30.1%				
AM Hopkins et al.	2020	International multicenter	IMvigor210: NCT02108652; IMvigor211: NCT02302807	Pooled post-hoc analnaysis of randomized controlled trials + single-arm trials	IMvigor210: 11 months; IMvigor211: 16 months	UC	847	Mixed lines	Within 30 days before to 30 days after the initiation of ICI	-	PD-L1	-
M Chalabi et al.	2020	International multicenter	POPLAR: NCT01903993; OAK: NCT02008227	Retrospective pooled analysis of randomized controlled trials	19.2 months	Metastatic NSCLC (previously treated)	757	Mostly≥ 2nd-line	Within 30 days before and 30 days after the start of first study treatment	POPLAR: 2013.8-2014.3; OAK: 2014.3-2015.4	PD-L1	Systemic antibiotics
PY Cren et al.	2020	France	-	Retrospective real-world study	8.7 months	Advanced melanoma	1585	Mixed lines	Within 2 months before the start of ICI to 1 month after the start of ICI	2012.1-2014.11	CTLA-4	-
U Swami et al.	2020	USA	-	Retrospective single-center cohort	18.46 months （0.89–62.52）	Advanced cutaneous melanoma	169	Mixed lines	Within 2 months prior to the initiation of PD-1 therapy	2012.8-2017.7	PD-1	-

V Serpas et al.	2020	USA	-	Single-center retrospective study	-	MSI-H mCRC	45	-	2 weeks before ICI to 6 weeks after ICI	-	PD-1+CTLA-4	-
A Castello et al.	2021	Italy	NCT03563482 and others	Retrospective analysis of prospective registration	12.4 months (9.7–15.2)	NSCLC	50	Mixed lines	Within 30 days before and 30 days after initiating ICI therapy	2015.12-2019.5	PD-1/PD-L1±CTLA-4	β-lactam±inhibitor, quinolone, rifaximin, glycopeptide
A Cortellini et al.	2021	European multicenter	-	Retrospective multicenter real-world study	21.8 months	NSCLC	950	1st-line	Within the 30 days before first-line treatment initiation	2017.1-2020.5	PD-1	-
AC Balado et al.	2021	Spain	-	Retrospective real-world study	-	Advanced/metastatic NSCLC	49	1st-line	Within ±30 days of pembrolizumab start	2017.7-2020.1	PD-1	-
K Rounis et al.	2021	Greece	-	Prospective observational study	6.37 months	mNSCLC	66	2nd-line	Within 30 days prior to ICI or within first 12 weeks after initiation	2017.11-2019. 11	PD-1+PD-L1	-
KS Cheung et al.	2021	Hong Kong, China	-	Retrospective cohort study	16.5 months (5.6–44.3)	HCC	395	-	Within 30 days before or after ICI initiation (±30 days window)	2014.1-2019.12	PD-1+CTLA-4	Penicillins, cephalosporins, macrolides, carbapenems, quinolones, tetracyclines, aminoglycosides, nitroimidazoles, glycopeptides, sulfa/trimethoprim, etc.

L Derosa et al.	2021	France	GETUG-AFU 26 NIVOREN；NCT0301335	Multicenter phase II clinical trial with retrospective subgroup analysis of ATB exposure	-	ccRCC	707	Mostly ≥ 2nd-line	Use of systemic antibiotics from 60 days before to 42 days after nivolumab initiation	2016.2-2017.6	PD-1	-
L Gaucher et al.	2021	France	PI2018_843_0062	Retrospective cohort study	-	Mixed tumors	372	-	-	2010.12-2019.12	PD-1+CTLA-4	β-lactams, macrolides, quinolones, glycopeptides, nitroimidazoles, tetracyclines, TMP-SMX, oxazolidinones, aminoglycosides, lipopeptides, streptogramins, etc.
LM Chambers et al.	2021	USA	-	Retrospective cohort study	9.5 months (4.6–19.9)	Recurrent gynecological cancers (endometrial cancer, cervical cancer, ovarian cancer)	101	-	Within 30 days before ICI and during ICI treatment	2017.1-2020.9	PD-1/PD-L1±CTLA-4	-

M Kostine et al.	2021	France	-	Retrospective study	-	Various tumors, predominantly melanoma, NSCLC, RCC	635	Mixed lines	Use within 1 month before or 1 month after ICI initiation	2015. 5-2017. 9	PD-1+PD-L1+CTLA-4	β-lactam 69%, Sulfonamide 17%, Quinolone 13%, Macrolide 12%, Aminoglycoside 7%, Tetracycline 5%
MJ Geum et al.	2021	Korea	-	Retrospective cohort study	-	Advanced/recurrent NSCLC	140	Mixed lines	From 30 days before nivolumab initiation until completion of nivolumab therapy	2015.7-2018.6	PD-1	Various systemic antibiotics, mainly broad-spectrum agents
N Ochi et al.	2021	Japan	-	Retrospective cohort study	-	NSCLC	531	-	Within 2 months before or 1 month after starting ICI	2015.12-2018.5	PD-1+PD-L1	β-lactams most common
P Fessas et al.	2021	International multicenter	-	Retrospective real-world study based on prospectively maintained cohort	17.0 months	HCC	449	Mixed lines	Within 30 days before to 30 days after the initiation of ICI	2017-2019	PD-1+PD-L1+CTLA-4	Mostly β-lactams, quinolones
PH Lu et al.	2021	Taiwan, China	-	Retrospective cohort study	-	NSCLC	340	Mixed lines	Within 30 days before ICI	2016.1-2019.3	PD-1+PD-L1±CTLA-4	Fluoroquinolones 39%, penicillins 38%, 1st/2nd-gen cephalosporins 33%, 3rd/4th-gen cephalosporins 30%, carbapenems 7%, macrolides 5%

Q Giordan et al.	2021	France	-	Retrospective study	10 months （5-12）	Multiple cancers (melanoma, NSCLC, UADT, RCC)	138	Mixed lines	Within 60 days prior to ICI initiation	2018.1-2019.12	PD-1± CTLA-4	β-lactams (55%), tetracyclines (31%), fluoroquinolones (8.6%)
S Buti et al.	2021	Italy		Retrospective cohort study	21.6 months	Advanced/metastatic solid cancers: NSCLC 70%, melanoma14.7%, RCC9.2%, others6%.	217	Mixed lines	Within 30 days before ICI	2014. 4-2019. 2	PD-1+PD-L1+CTLA-4	Systemic antibiotics
A Braun et al.	2022	USA	-	Retrospective real-world database study	-	Advanced RCC	1809	1st-line	Within ±3 months of ICI start	-	ICI	-
AF Nyein et al.	2022	USA	-	Retrospective single-center study	-	NSCLC (Stage III/IV)	256	-	From 60 days before ICI to 30 days after ICI	2011.1-2017.3	PD-1+PD-L1+CTLA-4	β-lactams, fluoroquinolones, macrolides, cephalosporins, tetracyclines; most commonly used: levofloxacin, cefazolin, azithromycin

H Qiu et al.	2022	China	-	Retrospective study	-	NSCLC	148	Mixed lines	From 60 days before to 60 days after first ICI cycle	2018.10-2021.6	PD-1+PD-L1	β-lactams, quinolones, and their combination; subclasses of β-lactams: penicillins, cephalosporins, carbapenems
I Joshi et al.	2022	USA	-	Retrospective study	505 days (16.6 months)	NSCLC	136	1st-line	1 month before ICI	2014.1-2019.5	PD-1	-
JR Wang et al.	2022	China	-	Retrospective cohort study	-	Primary liver cancer	215	1st-line or 2nd-line	Within ±30 days of immunotherapy initiation	2018.6-2020.10	PD-1	Cephalosporins, aminoglycosides, fluoroquinolones, and carbapenems
K Takada et al.	2022	Japan	-	Retrospective cohort study	49.1 months (95% CI 43.3-51.8)	Advanced NSCLC	293	Mostly≥ 2nd-line	Systemic antibiotics within 30 days before ICI initiation	2016.1-2018.9	PD-1	Systemic antibiotics
M Sun et al.	2022	China	-	Retrospective cohort study	-	NSCLC	315	1st-line or 2nd-line	Within 1 month before or 1 month after initiating ICI therapy	2015.1-2021.3	PD-1+PD-L1	Penicillins (most common, 66%) and cephalosporins (20%), among others
WA Stokes et al.	2022	USA	-	Nested cohort study	-	NSCLC	3634	-	30 days before ICI to 30 days after ICI	2010-2018	PD-1+PD-L1	Mostly β-lactams
A Kim et al.	2023	Korea	-	Retrospective cohort study	-	uHCC	177	1st-line	Early ATB: use within 30 days prior to ICI initiation; Late ATB:	2020. 8-2022.12	PD-L1	-

									at any time during ICI treatment occurring >30 days after initiation			
A Wada et al.	2023	Japan	-	Retrospective single-center study	17.2 months (14.4-26.8)	R/M HNSCC	110	Mixed lines	Within ±30 days of nivolumab initiation	2017. 5-2020. 3	PD-1	-
CG Kim et al.	2023	Korea	-	Retrospective exploratory cohort+retrospective validation+prospective validation	-	Advanced Gastric Cancer	152	Mixed lines	Within 28 days before ICI start	-	PD-1	-
GA Şen et al.	2023	Turkey	-	Retrospective cohort	-	Advanced NSCLC	90	1st-line or 2nd-line	Within 12 weeks after ICI initiation	-	PD-1+PD-L1	Moxifloxacin, clarithromycin, amoxicillin-clavulanate; piperacillin-tazobactam, meropenem, vancomycin, colistin, TMP-SMX etc.
HJ Choi et al.	2023	Korea	-	Retrospective dual-center study	-	Recurrent or metastatic gynecologic malignancies	215	Mixed lines	Within 60 days prior to ICI initiation	-	PD-1	Systemic antibiotics

K Maesaka et al.	2023	Japan	UMIN000034611	Retrospective multicenter analysis from prospectively registered cohorts	5.1 months (antibiotic+); 7.0 months (antibiotic-)	HCC	105	1st-line	Within 30 days before initiation of atezolizumab + bevacizumab	2020.10-2022. 1	PD-L1	Cefmetazole, sulbactam/cefoperazone, cefazolin, cefepime, cefpodoxime proxetil, sulbactam/ampicillin, tazobactam/piperacillin, amoxicillin/clavulanate, levofloxacin, teicoplanin, rifaximin
L Eng et al.	2023	Canada	-	Retrospective cohort study based on population	-	Multiple cancers (lung cancer 53%, melanoma 34%, others)	2,737	-	Two exposure windows: (1) Within 1 year prior to ICI initiation (2) Any ATB use within 60 days prior to ICI initiation	2012.6-2018.10	PD-1+CTLA-4	Fluoroquinolone, etc.
S Buti et al.	2023	Italy	-	Retrospective analysis of real-world prospective registry	16 months	mRCC	305	1st-line	Within 30 days before the initiation of ICI	2019.4-2019.10	PD-1+CTLA-4	-
T Hatanaka et al.	2023	Japan	UMIN000043219	Retrospective study	-	HCC	441	Mixed lines	Within ±30 days of immunotherapy	2020.9-2022.4	PD-1	Quinolones; Penicillin; Cephalosporins
Y Akashi et al.	2023	Japan	-	Retrospective cohort study	16.5months	Advanced/metastatic UC	41	-	Within 1 month before or after pembrolizumab, duration ≥1 week	2018.1-2021.12	PD-1	Cephalosporins 7, Fluoroquinolones 5, Penicillins 4

A Darwin et al.	2024	USA	-	Retrospective cohort	-	Melanoma, Stage IV	876	1st-line	6 months before and after ICI	2010.1-2020.3	PD-1+CTLA-4	Vancomycin, metronidazole, piperacillin–tazobactam
E Chorti et al.	2024	Germany	-	Retrospective multicenter cohort	21.3 months (8.5–35.5)	Unresectable stage III–IV melanoma	578	1st-line	pABT (within 60 days before ICB): 41; cABT (during ICB): 109; noABT: 428	2013.6-2018.9	PD-1±CTLA-4	Penicillins, quinolones, cephalosporins, some macrolides
F Deng et al.	2024	China	-	Retrospective cohort study	25.2 months (22.99–27.34)	NSCLC, locally advanced or metastatic	316	Mixed lines	Within 30 days before ICI initiation (p-ATB) or during ICI treatment	2018.1-2023.10	PD-1+PD-L1	β-lactams, quinolones, quinolones + β-lactams, macrolides
J Zhong et al.	2024	China	-	Retrospective cohort study	-	ES-SCLC	214	-	Antibiotic use from 2 months before to 2 months after first ICI	2019.7-2020.12	PD-1+PD-L1	Penicillins 41.7%, quinolones 22.9%, cephalosporins 22.8%, carbapenems 6.3%, glycopeptides 4.2%, aminoglycosides 2.1%, macrolides 2.1%
K Alshammari et al.	2024	Saudi Arabia	-	Retrospective cohort study	7.03months (mean)	HCC	59	Mixed lines	Within 30 days pre-ICI, during ICI, or 30 days post-ICI	-	PD-1	Ceftriaxone, Ciprofloxacin, Piperacillin/Tazobactam, Meropenem, Augmentin, Moxifloxacin
K Tamura et al.	2024	Japan	-	Retrospective study (Propensity	45.9 months (antibiotics+),	NSCLC	63	Mostly 1st-line	30 days before ICI	2018.12-2020.12	PD-1+PD-L1	AMPC/CVA, PIPC/TAZ, LVFX, ABPC/SBT, etc.

				Score Matching, PSM)	47.2 months (antibiiot ics-)							
M Metselaar-Albers et al.	2024	Netherlands	-	Observational single-center cohort	10.0 months	Melanoma+ NSCLC	1908	1st-line	Antibiotics within 30 days before ICI start	2011.1-2018.12	PD-1/PD-L1+CTLA-4	Systemic antibiotics
M Ueno et al.	2024	Japan	JCOG1808/ NCCH1817（SNIPE）	Phase I/II single-arm trial	6.4 months	Advanced biliary tract cancer	32	2nd-line	Within 1 month prior to nivolumab start	2019. 8-2020. 11	PD-1	-
N Katsurayama et al.	2024	Japan	-	Retrospective real-world study	15.4months	Advanced RCC	128	1st-line	Within 1 month prior to ICI initiation	2018-2023	PD-1+CTLA-4 或 PD-1/PD-L1+TKI	-
WC Luo et al.	2024	China	-	Prospective single-center observational cohort	-	Advanced NSCLC (EGFR+)	74	Mixed lines	2 months before and after ICI	2019.3-2022.9	PD-1+PD-L1	-
YF Xie et al.	2024	China	-	Retrospective cohort study	-	NSCLC accounted	631	-	Within 3 months before initiation of ICI therapy	2018.12-2023.6	ICI	Cephalosporins

						for 74.8% (≈472/631)						
A Rousseau et al.	2025	France	ATHENA cohort	Retrospective cohort study using target trial emulation	-	Advanced NSCLC	41,529	1st-line	From 60 days prior to and 42 days after pembrolizumab initiation	2015.1-2022.12	PD-1	Fluoroquinolones, macrolides, other β-lactams, penicillins, penicillins + β-lactamase inhibitors, sulfonamides, and several multi-antibiotic regimens, etc.
CB Xavier et al.	2025	USA	-	Retrospective cohort study based on early-phase clinical trials	-	Various solid tumors (CRC 24.5%, HNC 10.5%, PC 9.4%)	827	-	Any antibiotic administration within ±30 days of immunotherapy initiation	2014.6-2024.6	Experimental immunotherapy	-
C Zhang et al.	2025	China	-	Retrospective single-center study	-	Advanced biliary tract cancer	182	1st-line	30 days before ICI to 30 days after ICI	2018.10-2021.7	PD-1	-
F Nie et al.	2025	China	-	Retrospective single-center study	-	Multiple cancer types (lung cancer: 40 cases, gastric cancer: 25 cases, others: 69 cases)	134	-	Within 30 days before and after initiating ICI therapy	2021.1-2023.10	PD-1	Imipenem, levofloxacin, etc.

K Minohara et al.	2025	Japan	-	Retrospective cohort study	-	Recurrent/metastatic HNC	112	Mixed lines	Within 60 days before ICI	2017.6-2022.7	PD-1	β-lactam, cephalosporin, macrolides, tetracycline, quinolone, etc.
KYY Ng et al.	2025	Singapore	-	Retrospective study	25.1 months	HCC	168	Mixed lines	Within 30 days before and 30 days after initiating ICI therapy	2015.5-2019.11	ICI	-
N Ochi et al.	2025	Japan	-	Retrospective multicenter study	17.0 months (15.8–18.2)	NSCLC	451	1st-line	2 months before therapy to 1 month after therapy	2018.12-2020.12	PD-1+PD-L1	-
NB Balmaceda et al.	2025	USA	-	Retrospective cohort study	-	Advanced GC, EC, GEJ	990	1st-line	Within 42 days before or after ICI initiation	-	ICI	-
P Guerrero et al.	2025	Spain (Madrid)	-	Retrospective study	-	Multiple cancer types (NSCLC, UC, RCC, melanoma, HNC, etc.)	475	Mixed lines	From 60 days before to 42 days after first ICI cycle	2015.4-2022.10	PD-1+PD-L1+CTLA-4	β-lactams, quinolones, carbapenems, cephalosporins, macrolides, glycopeptides, etc.
T Hu et al.	2025	China	-	Retrospective single-center study	33.79 months	mNSCLC (Stage IV)	199	-	During ICI treatment	2017.12-2021.10	PD-1+PD-L1	β-lactams, quinolones, aminoglycosides etc.
Y Li et al.	2025	China	-	Retrospective cohort study	-	Advanced HCC	102	-	Within 60 days before or after ICI administration	2021.1.1-2023.12.30	ICI	Mostly β-lactams

S Bailey et al.	2026	USA	-	Retrospective single-center rstudy	-	Advanced, metastatic, or unresectable melanoma	214	-	During ICI and 30 days after ICI therapy	2014.6-2022.9	PD-1±CTLA-4/LAG-3	Antibiotics were classified as with or without anaerobic coverage.

Table S5. Subgroup analysis of clinical response of objective response rate (ORR) in the treatment of antibiotics with immune checkpoints inhibitors (ICIs)

Subgroup	No. of Studies	Random-effect Model		I^2
		OR [95% CI]	P Value	
**Total**	**31**	**0.60 [0.46, 0.77]**	**0.00**	**62.40%**
**Cancer Type**				
BTC	2	0.30 [0.14, 0.64]	0.00	0.00%
GC	1	0.14 [0.02, 1,13]	0.06	-
HCC	2	0.56 [0.18, 1.76]	0.32	22.90%
HNC	1	0.94 [0.38, 2.34]	0.90	-
Melanoma	1	0.09 [0.01, 1.61]	0.10	-
NSCLC	12	0.76 [0.59, 0.98]	0.03	29.62%
RCC	4	0.62 [0.38, 0.99]	0.05	6.66%
Multiple tumors				
**Line of Therapy**				
1-st line	6	0.61 [0.44, 0.84]	0.00	26.93%
1-st line or 2-nd line	3	0.42 [0.20, 0.90]	0.02	52.31%
2-nd line	2	0.52 [0.15, 1.82]	0.31	0.00%
≥2-nd line	2	1.01 [0.37, 2.75]	0.98	65.36%
Mixed lnes	12	0.62 [0.44, 0.88]	0.01	34.26%
-	6	0.59 [0.23, 1.55]	0.29	87.45%
**Immunotherapy**				
PD-1	9	0.62 [0.45, 0.84]	0.00	19.44%
PD-1+CTLA-4	4	0.31 [0.10, 1.03]	0.06	66.22%
PD-1+PD-L1	10	0.82 [0.51, 1.30]	0.39	69.94%
PD-1+PD-L1+CTLA-4	7	0.54 [0.41, 0.71]	0.00	0.00%
PD-L1	1	0.29 [0.06, 1.36]0.12	0.12	-
**Trial Type**				
Prospective study	5	0.53 [0.23, 1.20]	0.13	51.28%
Retrospective study	26	0.61 [0.46, 0.80]	0.00	65.23%

Table S6. Subgroup analysis of survival outcome of overall survival (OS) in the treatment of antibiotics with immune checkpoints inhibitors (ICIs)

Subgroup	No. of Studies	Random-effect Model		I^2
		HR [95% CI]	P Value	
**Total**	**66**	**1.56 [1.44 1.69]**	**0.00**	**91.33%**
**Cancer Type**				
ESCC	1	5.88 [2.55, 13.55]	0.00	-
GC	1	3.05 [2.36, 3.93]	0.00	-
Gynecologic malignancies	2	1.62 [0.80, 3.04]	0.13	55.90%
HCC	7	1.39 [1.11, 1.74]	0.00	43.15%
HNC	2	1.72 [1.19, 2.49]	0.00	0.00%
Melanoma	6	1.36 [1.07, 1.74]	0.01	62.90%
NSCLC	27	1.50 [1.32, 1.70]	0.00	79.70%
RCC	5	1.54 [1.31, 1.80]	0.00	0.00%
SCLC	1	1.46 [1.01, 2.11]	0.04	-
UC	2	1.45 [1.21, 1.74]	0.00	0.00%
Multiple tumors	12	1.62 [1.34, 1.95]	0.00	96.22%
**Line of Therapy**				
1-st line	13	1.38 [1.19, 1.61]	0.00	71.95%
1-st line or 2-nd line	3	2.17 [1.46, 3.23]	0.00	0.00%
2-nd line	1	1.35 [0.76, 2.41]	0.00	0.00%
≥2-nd line	5	1.48 [1.28, 1.72]	0.00	91.33%
Mixed lnes	26	1.74 [1.50, 2.02]	0.00	81.55%
-	18	1.42 [1.24, 1.64]	0.00	80.71%
**Immunotherapy**				
PD-1	20	1.68 [1.42, 1.99]	0.00	96.31%
PD-L1	4	1.44 [1.25, 1.64]	0.00	0.00%
CTLA-4	1	1.68 [1.30, 2.18]	0.00	-
PD-1+CTLA-4	7	1.39 [1.10, 1.74]	0.00	76.46%
PD-1+PD-L1	15	1.63 [1.25, 2.13]	0.00	85.42%
PD-1+PD-L1+CTLA-4	13	1.57 [1.31, 1.89]	0.00	65.21%
Experimental immunotherapy	1	1.54 [1.22, 1.95]	0.00	-
-	5	1.41 [1.21, 1.65]	0.00	0.00%
**Trial Type**				
Prospective Study	5	1.83 [1.30, 2.58]	0.00	71.83%
Retrospective Study	60	1.55 [1.42, 1.69]	0.00	91.63%
Nested cohort study	1	1.38 [1.27, 1.49]	0.00	-

Table S7. Subgroup analysis of survival outcome of progression-free survival (PFS) in the treatment of antibiotics with immune checkpoints inhibitors (ICIs)

Subgroup	No. of Studies	Random-effect Model		I^2
		HR [95% CI]	P Value	
**Total**	**48**	**1.50 [1.32, 1.70]**	**0.00**	**81.36%**
**Cancer Type**				
ESCC	1	5.11 [2.42, 10.80]	0.00	-
GC	1	3.24 [2.43, 4.33]	0.00	-
Gynecologic malignancies	1	0.96 [0.59, 1.55]	0.87	-
HCC	6	1.14 [0.86, 1.50]	0.38	68.83%
HNC	2	1.83 [1.31, 2.53]	0.00	0.00%
Melanoma	3	1.31 [0.62, 2.76]	0.48	82.31%
NSCLC	20	1.56 [1.24, 1.95]	0.00	81.93%
RCC	4	1.60 [1.18, 2.16]	0.00	71.08%
SCLC	1	1.47 [1.03, 2.09]	0.03	-
UC	2	1.24 [1.05, 1.45]	0.01	0.00%
Multiple tumors	7	1.50 [1.21, 1.86]	0.00	72.60%
**Line of Therapy**				
1-st line	8	1.64 [1.27, 2.10]	0.00	69.76%
1-st line or 2-nd line	3	1.56 [0.93, 2.60]	0.09	68.00%
2-nd line	1	1.66 [0.97, 2.83]	0.07	-
≥2-nd line	2	1.31 [0.94, 1.82]	0.11	11.41%
Mixed lnes	21	1.65 [1.35, 2.03]	0.00	85.65%
-	13	1.20 [0.96, 1.51]	0.11	77.13%
**Immunotherapy**				
PD-1	14	1.64 [1.30, 2.06]	0.00	75.39%
PD-L1	3	1.42 [1.03, 1.94]	0.03	33.46%
PD-1+CTLA-4	3	1.54 [0.69, 3.42]	0.29	89.05%
PD-1+PD-L1	15	1.58 [1.19, 2.10]	0.00	84.12%
PD-1+PD-L1+CTLA-4	8	1.50 [1.09, 2.07]	0.01	86.32%
Experimental immunotherapy	1	0.98 [0.80, 1.20]	0.88	-
-	4	1.29 [1.12, 1.47]	0.00	0.00%
**Trial Type**				
Prospective Study	4	1.29 [1.14, 1.47]	0.00	0.00%
Retrospective Study	44	1.50 [1.31, 1.72]	0.00	81.38%

Table S8. Detail characteristics of 15 probiotic cohort included in the meta-analysis

Author	Year	Country	Study name	Trial type	Median follow-up	Cancer Type	N	Line of Therapy	Time frame	Probiotic treatment	Immunotherapy	Control Group	Results (Probiotic group)
M Svaton et al.	2020	Czech Republic	-	National multicenter retrospective study	-	Advanced NSCLC	224	Mixed lines	2015-2019	Lactobacillus	PD-1	ICI only	Not significantly associated with ORR, OS or PFS
Y Tomita et al.	2020	Japan	-	Retrospective study	-	NSCLC	118	Mixed lines	2016.1-2019.5	CBM588	PD-1+PD-L1	ICI only	Better ORR, PFS, and OS
K Miura et al.	2021	Japan	-	Single-center retrospective study	-	Advanced NSCLC	300	Mixed lines	2016.1-2018.7	-	PD-1	ICI only	Not significantly associated with ORR or OS
K Takada et al.	2021	Japan	-	Retrospective study	432 days	NSCLC	294	Mixed lines	2016.1-2018.9	Bifidobacterium (BIOFERMIN and LAC-B), CBM, antibiotic-resistant lactic acid bacteria	PD-1	ICI only	Better PFS
N Dizman et al.	2022	US	NCT03829111	Single-center, open-label, randomized phase I trial	12.2 months	Metastatic renal cell carcinoma	29	1st-line	2019.4-2020.12	CBM588	PD-1+CTLA-4	ICI only	Better PFS and ORR
A Morita et al.	2024	Japan	-	Multicenter retrospective cohort study	-	Advanced NSCLC	927	Mixed lines	OLCSG-ID: 2015.12-2018.5; OLCSG-ICD:	CBM588	PD-1+PD-L1	ICI only	Better PFS, OS and ORR

									2018.12-2020.12				
H Ebrahimi et al.	2024	US	NCT05122546	Phase I, randomized, controlled study	15.9 months	Locally advanced or metastatic renal cell carcinoma	30	1st-line	-	CBM588	PD-1	ICI only	Better ORR
H Nakatsukasa et al.	2024	Japan	-	Retrospective study	-	Advanced or recurrent NSCLC	289	Mixed lines	2016.1–2021.12	Bio-three (Clostridium butyricum, Streptococcus faecalis, and Bacillus mesentericus)	PD-1+PD-L1	ICI only	Better PFS and OS
IG Oliva et al.	2024	US	NCT03817125	Phase Ib, randomized controlled study	-	Melanoma	14	-	2019.2-2022.3	SER-401 defined Firmicutes-enriched bacterial consortium	PD-1	Placebo antibiotic+placebo microbiome modulation+nivolumab	Not significantly associated with ORR, mPFS, or mOS
L Tong et al.	2024	China	-	Prospective, non-interventional, real-world cohort study	-	SCLC	59	Mixed lines	2021.6-2022.12	Commercially available oral probiotic supplements	PD-1+PD-L1	ICI only	Better PFS and ORR
Y Tomita et al.	2024	Japan	-	Retrospective observational cohort study	14.5 months (PFS)/ 5.5	Stage IV or recurrent metastatic NSCLC	100	Mostly 1st-line	2019.1-2022.7	CBM588	PD-1, PD-L1, PD-1+CTLA-4	ICI only	Better PFS and OS

					months (OS)								
Y Wang et al.	2024	China	-	Three-center observational retrospective study	-	Advanced digestive tract cancer	352	Mixed lines	2019.3-2022.7	-	PD-1	ICI only	Better PFS and ORR, but not significantly associated with OS
J Arima et al.	2025	Japan	-	Retrospective two-arm comparative study	12 months	Advanced or recurrent metastatic urothelial carcinoma	44	≥ 2nd-line	2018.1-2024.1	CBM588	PD-1	ICI only	Better PFS and OS
K Takada et al.	2025	Multinational multicenter	-	Pooled post hoc individual patient data analysis of five randomized controlled trials	13.9 months	Advanced NSCLC	2248	1-st line	-	Probiotic supplements	PD-L1	ICI only	Not significantly associated with PFS or OS
TF Zhang et al.	2025	China	ChiCTR2000032093	Single-arm, single-center, exploratory trial	12 months	Advanced Esophageal Squamous Cell Carcinoma	10	2nd-line	2020.5-2022.9	Lacticaselbacillus paracasei	PD-1	External historical control: camrelizumab monotherapy group from the ESCORT study	Better PFS

Table S9. Subgroup analysis of clinical response of objective response rate (ORR) in the treatment of probiotics with immune checkpoints inhibitors (ICIs)

Subgroup	No. of Studies	Fixed-effect Model		I^2
		OR [95% CI]	P Value	
**Total**	**8**	**1.95 [1.46, 2.62]**	**0.00**	**37.02%**
**Cancer Type**				
Digestive tract cancer	1	1.73 [0.98, 3.05]	0.06	0.00%
Melanoma	1	0.17 [0.02, 1.72]	0.13	100.00%
NSCLC	3	1.97 [1.33, 2.92]	0.00	0.00%
RCC	2	7.69 [2.12, 27.90]	0.00	0.00%
SCLC	1	1.76 [0.61, 5.09]	0.29	0.00%
**Line of Therapy**				
1st-line	2	7.69 [2.12, 27.90]	0.00	0.00%
Mixed lines	5	1.88 [1.38, 2.56]	0.00	0.00%
-	1	0.17 [0.02, 1.72]	0.13	100.00%
**Immunotherapy**				
PD-1	4	1.62 [1.01, 2.58]	0.04	65.42%
PD-1+CTLA-4	1	5.50 [0.91, 33.18]	0.06	0.00%
PD-1+PD-L1	3	2.10 [1.42, 3.09]	0.00	0.00%
**Trial Type**				
Prospective Study	4	2.26 [1.11, 4.60]	0.02	65.93%
Retrospective Study	4	1.89 [1.37, 2.61]	0.00	0.00%

Table S10. Subgroup analysis of survival outcome of overall survival (OS) in the treatment of probiotics with immune checkpoints inhibitors (ICIs)

Subgroup	No. of Studies	Random-effect Model		I^2
		HR [95% CI]	P Value	
**Total**	**10**	**0.56 [0.41, 0.78]**	**0.01**	**73.72%**
**Cancer Type**				
Digestive tract cancer	1	0.90 [0.64, 1.27]	0.55	-
ESCC	1	0.43 [0.16, 1.17]	0.10	-
NSCLC	7	0.57 [0.40, 0.81]	0.00	73.29%
UC	1	0.10 [0.02, 2.52]	0.01	-
**Line of Therapy**				
1st-line	2	0.68 [0.27, 1.66]	0.39	84.88%
2nd-line	1	0.43 [0.16, 1.17]	0.10	-
≥2nd-line	1	0.10 [0.02, 0.52]	0.00	-
Mixed lines	6	0.58 [0.42, 0.82]	0.00	67.51%
**Trial Type**				
Prospective Study	2	0.76 [0.34, 1.71]	0.51	62.23%
Retrospective Study	8	0.52 [0.37, 0.73]	0.00	68.27%
**Immunotherapy**				
PD-1	5	0.63 [0.42, 0.93]	0.02	54.13%
PD-L1	1	1.03 [0.74, 1.43]	0.86	-
PD-1+PD-L1	3	0.43 [0.24, 0.76]	0.00	62.47%
PD-1+PD-L1+CTLA-4	1	0.41 [0.22, 0.76]	0.00	-

Table S11. Subgroup analysis of survival outcome of progression-free survival (PFS) in the treatment of probiotics with immune checkpoints inhibitors (ICIs)

Subgroup	No. of Studies	Random-effect Model		I^2
		HR [95% CI]	P Value	
**Total**	**11**	**0.53 [0.38, 0.74]**	**0.00**	**82.38%**
**Cancer Type**				
Digestive tract cancer	1	0.70 [0.49, 0.99]	0.04	-
ESCC	1	0.29 [0.11, 0.79]	0.02	-
NSCLC	7	0.64 [0.47, 0.87]	0.01	77.94%
RCC	1	0.15 [0.05, 0.46]	0.00	-
UC	1	0.07 [0.02, 0.34]	0.00	-
**Line of Therapy**				
1st-line	3	0.51 [0.17, 1.51]	0.22	92.29%
2nd-line	1	0.29 [0.11, 0.79]	0.02	-
≥2nd-line	1	0.07 [0.02, 0.34]	0.00	-
Mixed lines	6	0.56 [0.47, 0.67]	0.00	25.13%
**Trial Type**				
Prospective Study	3	0.41 [0.12, 1.39]	0.15	86.17%
Retrospective Study	8	0.55 [0.47, 0.63]	0.00	9.01%
**Immunotherapy**				
PD-1	5	0.47 [0.24, 0.93]	0.03	87.24%
PD-L1	1	1.15 [0.86, 1.54]	0.35	-
PD-1+PD-L1	3	0.54 [0.42, 0.69]	0.00	9.71%
PD-1+CTLA-4	1	0.15 [0.05, 0.46]	0.00	-
PD-1+PD-L1+CTLA-4	1	0.55 [0.34, 0.88]	0.01	-

Table S12. Detail characteristics of 15 fecal microbiota transplantation (FMT) cohort included in the meta-analysis

Author	Year	Country	Study name	Trial type	Median follow-up	Cancer Type	N	Line of Therapy	Time frame	Donor	Recipients	FMT treatment	Immunotherapy	Control Group	Results (FMT)
D Davar et al.	2021	US	NCT03341143	Single-arm phase II clinical trial	7 months	PD-1-refractory metastatic melanoma	16	≥2nd-line	2018.6-2020.1	Anti-PD-1 responders	16	Colonoscopy	PD-1	No	Clinical benefit in a subset of PD-1-refractory metastatic melanoma patients
EN Baruch et al.	2021	Israel	NCT03353402	Phase I, single-arm clinical trial	-	Anti-PD-1-refractory metastatic melanoma	10	≥2nd-line	2018.6-2019.3	Anti-PD-1 complete responders	10	Colonoscopy+oral FMT capsules	PD-1	No	Objective responses in anti-PD-1-refractory metastatic melanoma
A Ullern et al.	2024	Norway	NCT05286294	Phase IIA, open-label, single-arm study	-	Multiple advanced solid tumors	9	≥2nd-line	-	ICI responders with>12 months response	9	Up to five FMT administrations: first two by colonoscopy, 3rd–5th by clyster/enema	ICI	No	ORR= 0%; 5/9 (56%) had SD; 4/9 (44%) had PD

EN Baruch et al.	2024	US	NCT04729322	Single-center phase II trial	-	Multiple metastatic anti-PD-1-refractory Microsatellite Instability-High cancers	15	-	-	Anti-PD-1 complete responders	15	Colonoscopy; 10 patients also received maintenance FMT capsules	PD-1	No	Clinical benefit in a subset of anti-PD-1-refractory MSI-H cancers
WS Zhao et al.	2024	China	RENMIN-215/ ChiCTR2100046768	Open-label, single-arm, phase II trial	34 months	Refractory microsatellite stable/ proficient mismatch repair metastatic colorectal cancer	114	≥3rd-line	2021.5-2023.5	Healthy donors	20	Oral FMT capsules	PD-1	Real-world control	Improved OS and numerically higher PFS/ORR
Y Kim et al.	2024	Korea	NCT04264975	Prospective, single-arm, single-center study	-	Unresectable or metastatic solid cancers refractory to anti-PD-1	13	≥2nd-line	2019.1-2020.8	Anti-PD-1 responders	13	Colonoscopy	PD-1	No	Clinical benefit in a subset of anti-PD-1-refractory advanced solid cancers
DK Hadi et al.	2025	Canada	NCT03772899	Multicenter, single-arm phase I safety trial	52.8 months	Advanced unresectable or metastatic melanoma	20	-	2019.6-2022	Healthy donors	20	Oral FMT capsules	PD-1	No	Encouraging ORR, mPFS, and mOS
Y Chu et al.	2025	China	-	Single-center, open-label, single-arm	-	Multiple end-stage malignant solid tumors	5	≥3rd-line	2019.2-2019.12	Healthy donors	5	Nasojejunal tube infusion of	PD-1	No	Preliminary anti-tumor activity

				exploratory clinical study								washed microbiota suspension			
Y Du et al.	2025	China	ChiCTR2100043472	Single-center, single-arm, prospective exploratory study	26.1 months	Advanced NSCLC	7	-	2021.5-2022.8	Healthy donors	7	Oral FMT capsules	PD-1	No	Preliminary clinical activity
H Huang et al.	2026	China	MR-36-22-010054	Prospective, single-arm, phase II study	37.0 months	Stage IIIB–IV advanced NSCLC with secondary PD-1 resistance	27	Mixed lines	2021.10-2023.12	Healthy pediatric donors	27	Oral FMT capsules	PD-1	No	Encouraging activity
J Ma et al.	2026	China	ChiCTR2200058471	Prospective, single-arm, phase II trial	-	Unresectable hepatocellular carcinoma	37	≥3rd-line	2022.8-2025.3	-	13	Oral FMT capsules	PD-1	No	A certain role in disease control
R Fernandes et al.	2026	Canada	PERFORM Study, NCT04163289	Open-label, single-center, phase I trial	21.9 months	Metastatic/advanced renal cell carcinoma	20	1st-line	2020.2-2023.11	Healthy donors	20	Oral FMT capsules	PD-1/PD-1+CTLA-4	No	Preliminary efficacy in mRCC
S Duttagupta et al.	2026	Canada	FMT-LUMINate,	Multicenter, open-label, phase II trial	24 months	Advanced NSCLC and cutaneous melanoma	40	1st-line	2021.11-2024.3	Healthy donors	40	Oral FMT capsules	NSCLC: PD-1; melano	No	High ORR in both cohorts

			NCT04951583										ma: PD-1+CTLA-4		
S Porcari et al.	2026	Italy	TACITO study, NCT04758507	Randomized, phase II	32 months	Metastatic renal cell carcinoma	50	1st-line	2021.11-2023.11	An mRCC patient with complete and long-lasting response to ICIs	25	Colon infusion; oral FMT capsules	PD-1	Placebo FMT+ICI	Better PFS and ORR, numerically longer OS
Y Zhang et al.	2026	China	NCT04130763	Open-label, single-arm, single-center, phase I study	-	ICI-refractory Microsatellite Stable gastrointestinal cancer, mainly gastric cancer	10	≥2nd-line	2020.1-2022.9	Healthy donors	10	Oral FMT capsules	PD-1	No	Preliminary efficacy

Table S13. Subgroup analysis of clinical response of objective response rate (ORR) in the treatment of fecal microbiota transplantation (FMT) with immune checkpoints inhibitors (ICIs)

Subgroup	No. of Studies	ORR [95% CI]	P Value	I^2
**Total**	**16**	0.30 [0.16, 0.45]	0.00	81.50%
**Cancer Type**				
NSCLC	3	0.43 [0.08, 0.82]	0.00	86.99%
Melanoma	4	0.49 [0.22, 0.75]	0.00	78.10%
RCC	2	0.51 [0.36, 0.67]	0.00	0.00%
CRC	1	0.20 [0.05, 0.41]	0.00	-
HCC	1	0.00 [0.00, 0.13]	1.00	-
Gastrointestinal cancer	1	0.20 [0.01, 0.51]	0.03	-
Multiple tumors	4	0.07 [0.00, 0.22]	0.05	27.51%
**Line of Therapy**				
1st-line	4	0.65 [0.49, 0.79]	0.0	49.78%
≥2nd-Line	5	0.14 [0.04, 0.26]	0.00	17.33%
>3rd-line	3	0.13 [0.00, 0.42]	0.09	68.24%
Mixed Lines	1	0.30 [0.14, 0.48]	0.00	-
-	3	0.27 [0.00, 0.72]	0.05	86.59%
**Immunotherapy**				
PD-1	12	0.23 [0.12, 0.37]	0.00	68.99%
PD-1+CTLA-4	3	0.69 [0.51, 0.85]	0.00	50.39%
-	1	0.00 [0.00, 0.18]	1.0	-
**Trial Type**				
Single-arm	15	0.31 [0.16, 0.47]	0.00	82.27%
Dual-arm	1	0.20 [0.05, 0.41]	0.00	-
**Donors**				
Healthy donors	9	0.45 [0.28, 0.63]	0.00	76.05%
Responders	6	0.17 [0.04, 0.36]	0.00	71.40%
-	1	0.00 [0.00, 0.13]	1.00	-
**Route of administration**				
Oral FMT capsules	8	0.40 [0.19, 0.63]	0.00	86.21%
Colonoscopy	3	0.09 [0.00, 0.23]	0.03	20.78%
Both	4	0.27 [0.07, 0.51]	0.00	69.20%
Nasojejunal tube	1	0.40 [0.02, 0.86]]	0.02	-
